# Amino Acid Directed Helical Assemblies of Natural Berberine for Chirality‐Dependent Photodynamic Antibacterial Therapy

**DOI:** 10.1002/advs.202514096

**Published:** 2025-11-19

**Authors:** Haoqiang Zhao, Jingyi Jiao, Zhixia Wang, Chengyu Shi, Xinran Huang, Yixuan Lin, Haolin Yang, Haimin Lei, Guanghui Ouyang, Minghua Liu

**Affiliations:** ^1^ School of Chinese Pharmacy Beijing University of Chinese Medicine Beijing 102488 China; ^2^ Beijing National Laboratory of Molecular Sciences and CAS Key Laboratory of Colloid Interface and Thermodynamics Institute of Chemistry Chinese Academy of Sciences ZhongGuanCun North First Street 2 Beijing 100190 China

**Keywords:** antibacterial activity, berberine, nanostructures, self‐assembly, supramolecular chirality

## Abstract

While the biological effects of chiral pharmaceuticals at molecular level have been extensively studied, research remains limited on how chiral materials derived from natural herbal components exert biomedical effects at higher hierarchical levels. Herein, it is reported that helical nanostructures co‐assembled from a traditional Chinese medicine‐derived berberine (BBR) and an amino acid derivative (Fmoc‐Y) exhibit supramolecular‐chirality‐dependent photodynamic antibacterial activity. The inherent molecular chirality of the Fmoc‐Y component directs the organization of achiral BBR into chiral nanofibers. The experimental results reveal that supramolecular chirality governs nanofiber adsorption on bacterial surfaces and subsequent membrane penetration. Compared with left‐handed nanofibers, the chirality‐matched right‐handed ones enable more efficient intracellular delivery of BBR and reactive oxygen species (ROS) generation, leading to superior bacterial inhibition. This work highlights, for the first time, the unique role of supramolecular chirality in biomedicine applications based on natural herbal components, demonstrating how supramolecular chiral engineering synergizes with natural pharmacophores to develop high‐efficiency antimicrobial systems and advanced theranostic platforms.

## Graphical abstract

Helical nanostructures co‐assembled from berberine (BBR) and Fmoc‐Y display supramolecular‐chirality‐dependent photodynamic antibacterial activity. The molecular chirality of Fmoc‐Y organizes achiral BBR into chiral nanofibers, where supramolecular handedness controls bacterial surface adsorption, membrane penetration, intracellular BBR delivery, and ROS generation. Chirality‐matched right‐handed fibers exhibit markedly enhanced bacterial inhibition over left‐handed counterparts.

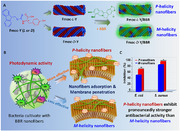

## 1 Introduction

Chirality, as a fundamental property of matter, plays a critical role in biomolecular recognition and pharmacological mechanisms.^[^ ^1^, ^2^, ^3^ ^]^ In pharmaceutical research, stereochemical differences of chiral molecules can lead to significant variations in pharmacological activity, metabolic pathways, and toxicity profiles due to their stereoselective interactions with chiral biological targets composed of chiral biological macromolecules including proteins, polysaccharides, and DNA.^[^ ^4^, ^5^, ^6^ ^]^ These characteristics have established chiral chemistry as a fundamental theoretical framework for drug design. While chiral chemistry underpins modern drug design paradigms at molecular level, herbal‐based traditional medicines (e.g., traditional Chinese medicine/TCM) present a more complex scenario. The canonical boiling treatment of TCM generally triggers components combination through synergistic non‐covalent interactions, leading to the formation of self‐assembled nano/micro structures and even larger aggregates. These structures might exhibit supramolecular chirality through assembly‐induced chirality transfer considering that more than a half of TCM bioactive components demonstrate molecular chirality.^[^ ^7^, ^8^, ^9^, ^10^, ^11^, ^12^ ^]^ Although researchers reported that some artificial self‐assembled chiral systems showed interesting chirality‐dependent cell adhesion and proliferation, protein adsorption, drug delivery, antibacterial adhesion, and cancer therapy,^[^ ^13^, ^14^, ^15^, ^16^, ^17^, ^18^, ^19^, ^20^ ^]^ the role of supramolecular chirality in the therapeutic effect of TCM has not yet been reported. Understanding the role of supramolecular chirality in mediating TCM's systemic therapeutic holds critical significance for deciphering their pharmacological mechanisms and comprehending the multilevel biological effects of chirality. The difficulty in solving this problem lies in two aspects. On the one hand, many natural TCM components only have one enantiomer, the synthesis of the other enantiomer is generally difficult, which makes it difficult to compare a group of materials with opposite chirality.^[^ ^21^ ^]^ On the other hand, current research predominantly focuses on chiral recognition mechanisms at the molecular level, while the regulatory effects of supramolecular chiral materials through self‐assembled ordered structures on biological systems remains inadequately explored.

**Figure 1:**
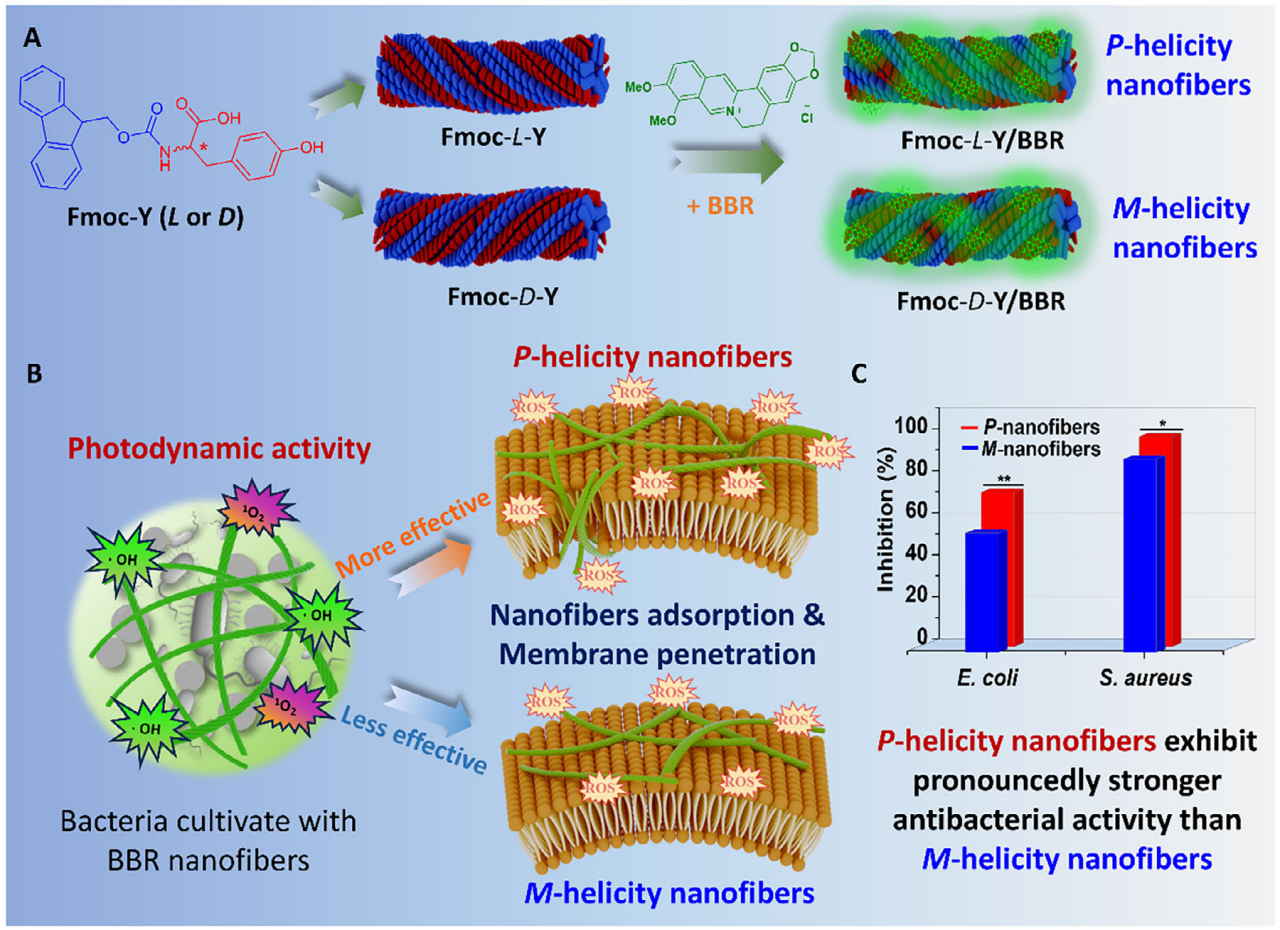
Chiral preference‐driven antibacterial performance of Fmoc‐Y/BBR co‐assembled systems. A) The chemical structures of Fmoc‐Y enantiomers and BBR, and schematic illustration of their co‐assembly into helicity‐controlled nanofibers. B) The photodynamic properties of nanofibers, and the differential interaction mechanisms between bacterial membranes and nanofibers with different supramolecular chirality. C) Comparison of photodynamic bacterial inhibition rates of *P*‐helicity nanofibers and *M*‐helicity nanofibers toward *E. coli* and *S. aureus*. ^*^ *p* < 0.05 and ^**^ *p* < 0.01.

Berberine (BBR), a cationic isoquinoline alkaloid derived from *Coptis chinensis*, exhibits broad‐spectrum antibacterial activity while demonstrating unique photophysical properties and self‐assembly potential owing to its planar structure and positive charge.^[^ ^22^, ^23^, ^24^, ^25^, ^26^, ^27^, ^28^, ^29^, ^30^, ^31^ ^]^ Its dual role as both a bioactive agent and a supramolecular building block makes it an ideal candidate to investigate the self‐assembly mechanisms of TCM components and their functional modulation of biological targets. Motivated by our ongoing investigations in the fields of chiral self‐assembly and TCM biomedicines,^[^ ^23^, ^24^, ^26^, ^32^, ^33^, ^34^, ^35^, ^36^, ^37^, ^38^, ^39^, ^40^, ^41^, ^42^, ^43^, ^44^, ^45^ ^]^ we herein report the construction of a chiral co‐assembled system using the chiral template compound 9‐Fluorenylmethyloxycarbonyl‐tyrosine (Fmoc‐Y and BBR to elucidate the role of supramolecular chirality in regulating photodynamic antibacterial therapy (**Figure** **1**). The commercially available amino acid‐derived Fmoc‐Y enantiomers, Fmoc‐*L*‐Y and Fmoc‐*D*‐Y, are capable of self‐assembling into chirality‐controlled nanofibers in alkaline PBS, providing ideal biocompatible nanoplatforms.^[^ ^46^, ^47^, ^48^, ^49^, ^50^ ^]^ By co‐assembling the achiral BBR with Fmoc‐Y enantiomers, the composite assemblies showed unambiguous handedness determined by the original Fmoc‐Y nanofibers. The induced circular dichroism (CD) bands and circularly polarized luminescence (CPL) activities of BBR confirmed efficient chirality transfer from the chiral nanofiber templates to the originally achiral BBR, establishing a foundation for studies on chiral TCM systems. These chiral nanofibers subsequently engaged in stereoselective interactions with cells featuring chiral biological targets, including proteins, phospholipids, and polysaccharides. Scanning electron microscopy (SEM) observations revealed that right‐handed nanofibers formed by Fmoc‐*L*‐Y/BBR exhibited more effective bacterial surface adsorption and subsequent membrane penetration, likely attributed to a supramolecular chirality‐matching principle. Under photodynamic conditions, Fmoc‐*L*‐Y/BBR assembly achieved 74.5% and 100% inhibition rates against *E. coli* and *S. aureus*, respectively, outperforming both BBR monomers and *D*‐handedness counterparts, representing the first example of supramolecular‐chirality‐dependent photodynamic antibacterial therapy system based on TCM components.

## 2 Results and Discussion

### 2.1 Co‐Assembly of Fmoc‐Y/BBR into Helical Nanofibers

**Figure 2:**
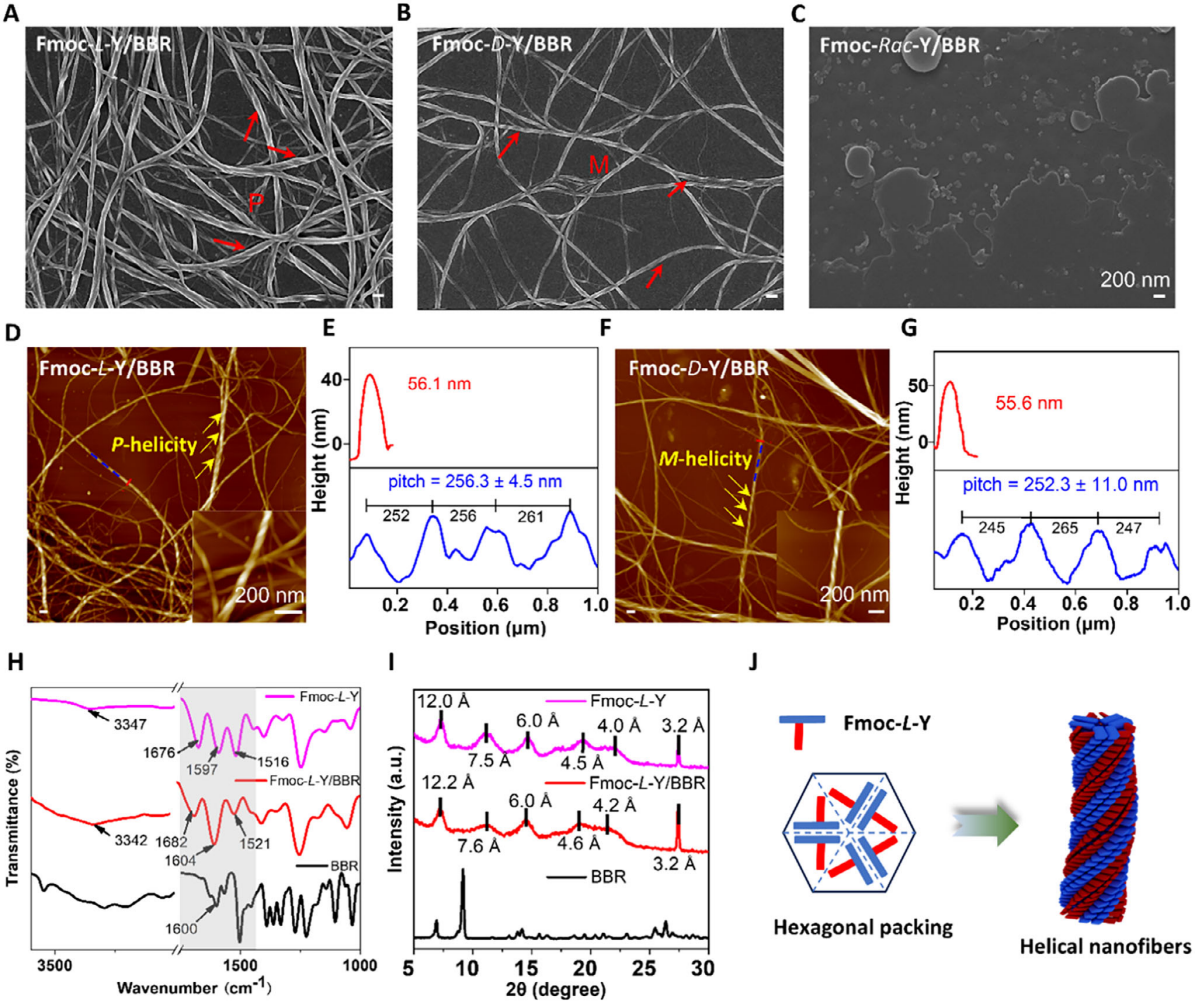
Morphology observation and packing mode studies. SEM images of A) Fmoc‐*L*‐Y/BBR co‐assembly, B) Fmoc‐*D*‐Y/BBR co‐assembly and C) Fmoc‐*Rac*‐Y/BBR. Scale bars: 200 nm. *P* and *M* indicate right‐handedness and left‐handedness of the obtained nanofibers, respectively. AFM images and height profiles of D,E) Fmoc‐*L*‐Y/BBR co‐assembly and F,G) Fmoc‐*D*‐Y/BBR co‐assembly. Insets showed local magnification. Scale bar: 200 nm. The red and blue lines in the AFM images indicated selected regions for height profile analysis. Values are expressed as the means ± SD (n = 3). (H) FT‐IR spectra and I) PXRD patterns of BBR powders (black line), Fmoc‐*L*‐Y assembly (pink line) and Fmoc‐*L*‐Y/BBR co‐assembly (red line). J) Proposed assembly modes. All the nanofibers were prepared in PBS, [Fmoc‐Y] = 34.7 mm, Fmoc‐Y/BBR = 29:1 in molar ratio.

Chirality transfer represents a pivotal process in the field of supramolecular chirality research. It facilitates the propagation of molecular‐level asymmetry to nanoscale architectures, providing critical insights for the design of chiral functional materials.^[^ ^33^, ^51^, ^52^, ^53^ ^]^ To elucidate how molecular chirality regulates the stereochemical assembly of TCM, we investigated the co‐assembly of Fmoc‐Y enantiomers (Fmoc‐*L*‐Y, Fmoc‐*D*‐Y) and their racemic mixture with BBR. Using a typical heating‐cooling assembly protocol in alkaline PBS, Fmoc‐Y enantiomers self‐assembled into hydrogels composed of helical nanofibers with opposite chirality, as shown by SEM images (Figure S1, Supporting Information). SEM observations indicated that upon co‐assembly with BBR, the stereospecific fiber architectures were maintained. Fmoc‐*L*‐Y/BBR formed right‐handed (*P*‐helicity) helical fibers, while Fmoc‐*D*‐Y/BBR assembled into left‐handed (*M*‐helicity) ones (**Figure** **2**A,B). In stark contrast, co‐assembly of racemic Fmoc‐Y (Fmoc‐*Rac*‐Y) with BBR yielded irregular aggregates lacking obvious helicity (Figure 2C). Atomic force microscopy (AFM) images corroborated the helical morphologies of the co‐assemblies and further revealed helical parameters such as fiber height and helical pitch. The height of Fmoc‐Y/BBR nanofibers was measured at ≈56 nm, and their helical pitch was determined to be ≈250 nm from AFM profiles (Figure 2D–G).

To elucidate the molecular packing modes within the helical nanofibers, we employed ultraviolet‐visible spectroscopy (UV–vis), fourier transform infrared spectroscopy (FT‐IR), powder X‐ray diffraction (PXRD), and nuclear magnetic resonance (NMR) techniques for characterization (Figure 2H,I; Figures S2–S5, Supporting Information). The FT‐IR spectrum of Fmoc‐Y nanofibers exhibited a characteristic N─H/O─H stretching vibration band near 3347 cm^−1^ and a carboxylate C═O stretching band at 1676 cm^−1^. The presence of amide I and II bands at 1597 and 1516 cm^−1^, respectively, confirmed the formation of amide hydrogen bonds. The IR peak profiles of Fmoc‐Y/BBR nanofibers were largely congruent with those of Fmoc‐Y nanofibers, suggesting that BBR likely adsorbed onto the fiber surface without disrupting Fmoc‐Y packing modes. However, notable wavenumber shifts of the amide I and II bands from 1597 and 1516 cm^−1^ to 1604 and 1521 cm^−1^, respectively, indicated weakened amide hydrogen bonding. Additionally, the carboxylate C═O stretching band shifted from 1676 to 1682 cm^−1^ (Figure 2H). Collectively, these results supported effective intermolecular interactions between Fmoc‐Y and BBR components. ^1^H‐NMR spectroscopy further corroborated these findings (Figure S5, Supporting Information). In the presence of an equimolar amount of BBR, the carboxylic and phenol protons of Fmoc‐*L*‐Y nearly vanished, implying intermolecular electrostatic complexation between anionic Fmoc‐Y and cationic BBR.

The diffraction peaks of Fmoc‐*L*‐Y nanofibers showed 2θ values of 12.0, 7.5, 6.0, 4.5, 4.0 and 3.2 Å. These values were corresponding to calculated *d*‐spacing ratios of 1, 1/√3, 1/2, 1/√7, 1/3 and 1/√12, unambiguously indicative of a hexagonal packing mode for molecular ordering.^[^ ^54^, ^55^, ^56^ ^]^ The PXRD pattern of BBR powder displayed sharp peaks characteristic of its crystalline nature. In contrast, the diffraction profile of Fmoc‐*L*‐Y/BBR co‐assembled nanofibers retained all fundamental features of the parent Fmoc‐*L*‐Y template, confirming that BBR incorporation did not disrupt the hexagonal packing arrangement—a finding consistent with FT‐IR results (Figure 2I; Figure S3, Supporting Information). Integrating these characterizations, we propose the following assembly mechanism (Figure 2J): Cooperative *π*–*π* stacking and intermolecular hydrogen bonding drive T‐shaped Fmoc‐*L*‐Y molecules to adopt a hexagonal packing geometry, nucleating chiral supramolecular structures that propagate into helical nanofibers. This hierarchical assembly process facilitates chirality transfer from molecular to nanoscale dimensions, manifesting as supramolecular chirality detectable via both morphological and chiroptical analyses. In the co‐assembled system, BBR molecules are hypothesized to adsorb onto the fiber surface through electrostatic interactions and *π*–*π* stacking (Figure 1A). The helical topology of the nanofiber surface imposes asymmetric steric constraints on BBR guests, inducing their chiral alignment with the underlying template.

### 2.2 Optical and Chiroptical Properties of Fmoc‐Y/BBR Nanofibers

**Figure 3:**
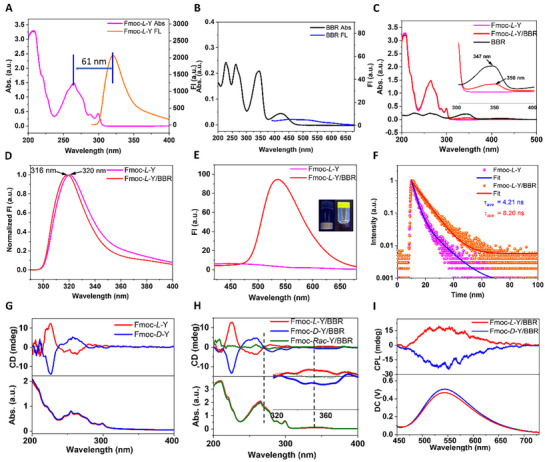
Optical properties of the nanofibers. A) Absorption spectrum (pink line) and emission spectra (λ_ex_ = 271 nm, orange line) of Fmoc‐*L*‐Y nanofibers. B) Absorption spectrum (black line) and emission spectra (λ_ex_ = 390 nm, blue line) of BBR in deionized water, [BBR] = 1.34 mm. C) UV–vis absorption spectra of BBR water solution (black line), Fmoc‐*L*‐Y nanofibers (pink line) and Fmoc‐*L*‐Y/BBR nanofibers (red line). D) Normalized FL spectra of Fmoc‐*L*‐Y nanofibers (pink line) and Fmoc‐*L*‐Y/BBR nanofibers (red line) in UV region, λ_ex_ = 271 nm. E) FL spectra of Fmoc‐*L*‐Y nanofibers (pink line) and Fmoc‐*L*‐Y/BBR nanofibers (red line) in visible light region, λ_ex_ = 390 nm. F) Fluorescence lifetime of Fmoc‐*L*‐Y nanofibers (blue line, monitored at 320 nm) and Fmoc‐*L*‐Y/BBR nanofibers (red line, monitored at 550 nm). G) CD spectra of Fmoc‐*L*‐Y (red line) and Fmoc‐*D*‐Y (blue line) nanofibers. H) CD spectra of Fmoc‐*L*‐Y/BBR (red line), Fmoc‐*D*‐Y/BBR (blue line) and Fmoc‐*Rac*‐Y/BBR (green line) in PBS. I) CPL spectra of assemblies of Fmoc‐*L*‐Y/BBR (red line) and Fmoc‐*D*‐Y/BBR (blue line) in PBS (PH =8.3)/ DI water =1/1 (λ_ex_ = 390 nm). All the nanofibers were prepared in PBS, [Fmoc‐Y] = 34.7 mM, Fmoc‐Y/BBR = 29:1 in molar ratio.

The optical and particularly chiroptical properties of the synthesized helical nanofibers are crucial for their functional applications. Initially, we characterized the optical behavior of BBR monomers and Fmoc‐*L*‐Y self‐assembly. The Fmoc‐*L*‐Y nanofibers exhibited an absorption maximum at 265 nm, attributed to *π*–*π* ^*^ transitions of the Fmoc chromophores (**Figure** **3**A; Figure S2, Supporting Information). Upon co‐assembly with BBR, the absorption maximum of BBR shifted from 347 to 350 nm, indicative of weak electronic coupling between the Fmoc and BBR chromophores (Figure 3C). The emission spectrum of Fmoc‐*L*‐Y nanofibers showed a broad band centered at 320 nm, whereas aqueous BBR monomers displayed no detectable emission under photoexcitation (Figure 3B, λ_ex_ = 390 nm). Co‐assembly markedly altered these emission profiles. Under Fmoc‐specific excitation (λ_ex_ = 271 nm),^[^ ^50^ ^]^ the emission band of the Fmoc chromophore blue‐shifted from 320 to 316 nm (Figure 3D). Conversely, excitation at BBR's absorption maximum (Figure S6, Supporting Information, λ_ex_ = 390 nm) generated a strong emission band at ≈550 nm (Figure 3E), indicating an aggregation‐induced emission (AIE) phenomenon of BBR (Figure S7, Supporting Information). This behavior was rationalized by restricted intramolecular motion of BBR molecules adsorbed onto the helical nanofibers. The confinement effect further increased the fluorescence quantum yield to 65.27% (Figure S8, Supporting Information) and extended the fluorescence lifetime from 4.21 to 8.20 ns (Figure 3F).

Subsequently, we characterized the chiroptical properties of the chiral assemblies. CD spectra of single‐component Fmoc‐(*L/D*)‐Y self‐assembly exhibited characteristic signals in the 200–300 nm range (Figure 3G). Co‐assembly with BBR preserved the intrinsic Cotton effects of Fmoc‐Y self‐assemblies, while a newly induced CD band spanning 320–380 nm revealed successful chirality transfer from the helical nanofibers to adsorbed BBR molecules (Figure 3H). Notably, co‐assembly of racemic Fmoc‐Y with BBR showed negligible CD activity. These results demonstrate that the supramolecular chirality in these systems originates from molecular chirality of Fmoc‐Y coupled with assembly‐mediated chirality transfer mechanisms. CPL serves as another powerful tool for probing chirality in photofunctional materials.^[^ ^32^, ^33^, ^34^, ^35^, ^40^ ^]^ Given the observed CD activity and strong emission of the co‐assembled nanofibers, we successfully detected mirror‐image CPL signals from Fmoc‐*L*‐Y/BBR and Fmoc‐*D*‐Y/BBR nanofibers (Figure 3I), with maximum luminescent dissymmetry factors of ±3.0 × 10^−3^ (Figure S9, Supporting Information).

### 2.3 Photodynamic Effects and Biocompatibility

**Figure 4:**
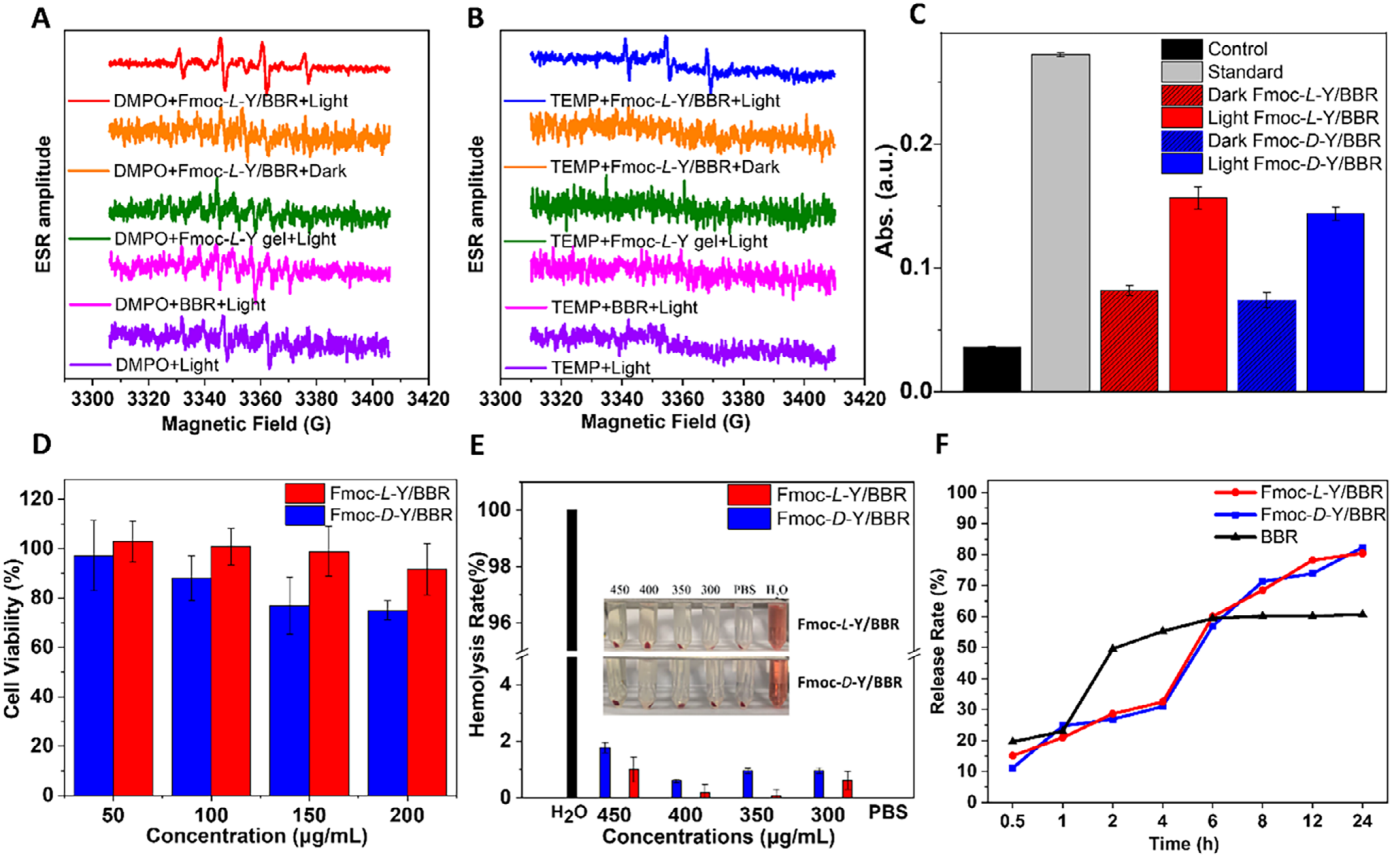
Photodynamic properties, biocompatibility and sustained‐release characteristics of Fmoc‐Y/BBR nanofibers. A) ESR signals of DMPO solution after white light irradiation (purple line), DMPO & BBR mixture after white light irradiation (pink line), DMPO & Fmoc‐*L*‐Y self‐assembly mixture after white light irradiation (green line), DMPO & Fmoc‐*L*‐Y/BBR co‐assembly mixture under dark conditions (orange line), DMPO & Fmoc‐*L*‐Y/BBR co‐assembly mixture after white light irradiation (red line). [DMPO] = 2.0 mm B) ESR signals of 4‐oxo‐TEMP solution after white light irradiation (purple line), 4‐oxo‐TEMP & BBR mixture after white light irradiation (pink line), 4‐oxo‐TEMP & Fmoc‐*L*‐Y self‐assembly mixture after white light irradiation (green line), 4‐oxo‐TEMP & Fmoc‐*L*‐Y/BBR co‐assembly mixture under dark conditions (orange line), 4‐oxo‐TEMP & Fmoc‐*L*‐Y/BBR co‐assembly mixture after white light irradiation (blue line). [TEMP] = 2.0 mm C) Absorption intensity of Griess reagent in the hydroxyl radical detection kit demonstrated a positive correlation with the amount of hydroxyl radicals formed, where higher absorption intensity indicates greater radical generation capacity. λ_em_ = 550 nm. D) Cell viability treated with two chiral assemblies at 24 h. E) The hemolytic test of two chiral assemblies at different concentrations. F) Drug release profile of Fmoc‐Y/BBR chiral hydrogels. All the nanofibers were prepared in PBS, [Fmoc‐Y] = 34.7 mM, Fmoc‐Y/BBR = 29:1 in molar ratio. Values are expressed as the means ± SD (n = 3).

The co‐assembled helical nanofibers effectively restricted intramolecular motion of BBR, thereby significantly enhancing fluorescence while generating singlet oxygen and hydroxyl free radicals. To this end, we conducted a thorough investigation into the PDT potential of the Fmoc‐Y/BBR chiral nanofibers using electron spin resonance (ESR) spectroscopy (**Figure** **4**A,B; Figure S10, Supporting Information). In this study, we utilized 5,5‐dimethyl‐1‐pyrroline‐*N*‐oxide (DMPO) and 2,2,6,6‐Tetramethyl‐4‐piperidone hydrochloride (4‐oxo‐TEMP) as specific radical scavengers for hydroxyl radicals (•OH) and singlet oxygen (^1^O_2_), respectively, to ascertain the types of reactive oxygen species (ROS) produced by the chiral assemblies.^[^ ^57^ ^]^ Upon white light irradiation of the Fmoc‐*L*‐Y/BBR chiral assembly, the ESR signal of DMPO‐OH intensified, indicating an increase in hydroxyl radical production, with a distinct four‐peak signal characteristic emerging (Figure 4A, red curves). Furthermore, a clear triple‐peak signal with uniform intensity was observed, signifying the formation of ^1^O_2_ under white light exposure (Figure 4B, blue curves). Due to the overwhelming presence of •OH radicals and the relatively lower reactivity of dissolved superoxide anion (•O_2−_) radicals with DMPO in an aqueous environment, we decided against pursuing the investigation into their potential release of •O_2−_ radicals. We further used the •OH detection kit to test the ability of the Fmoc‐Y/BBR chiral assembly to generate •OH under the driving of PDT. The results illustrated that the absorbance of the Fmoc‐Y/BBR under white light were significantly higher than that under dark conditions, confirming that BBR can photolyze water to generate •OH through the Type I photodynamic pathway (Figure 4C). Moreover, the absorbance of the *L*‐form assembly under illumination was slightly higher than that of the *D*‐form (Φ_L_ = 44.99 vs Ф_D_ = 40.26 U mL^−1^). Our experimental findings confirmed that the Fmoc‐*L*‐Y/BBR co‐assembly effectively generated both •OH radicals and ^1^O_2_ upon white light irradiation, presenting a promising research pathway and potential application in the field of PDT.

Biosafety evaluation serves as a critical prerequisite for the clinical translation of therapeutic materials. To systematically assess the biocompatibility profile of Fmoc‐Y/BBR chiral assemblies, we performed comprehensive in vitro analyses including cytocompatibility and hemocompatibility evaluations. The chiral hydrogel exhibited good biosafety in cytotoxicity assessments with HaCat keratinocytes. The *L*‐configured assembly showed high cell viability (≈92% at 200 µg mL^−1^), while the *D*‐configured assembly yielded a viability of ≈76% under the same conditions (Figure 4D). Hemolytic evaluation demonstrated exceptional blood compatibility for both assemblies, with hemolysis rates maintained below 2% at 0.45 mg mL^−1^, well below the clinically acceptable 5% threshold, thereby meeting critical requirements for endovascular circulation applications (Figure 4E). Collectively, these findings establish the *L*‐formulated assembly as particularly promising for clinical development, owing to their optimal combination of minimal cytotoxicity and favorable hemocompatibility.

The great properties of the chiral nanofiber‐based hydrogel were comprehensively demonstrated through multiple characterization methods, including inversion tests, SEM, rheological measurements, and Zeta potential analysis (Figures S11–S14, Supporting Information). In controlled‐release studies, the chiral hydrogels exhibited improved drug release kinetics when compared with free BBR (Figure 4F). Whereas free BBR showed rapid early‐phase release (60.72% cumulative release at 6 h), the Fmoc‐*L*‐Y/BBR hydrogel demonstrated sustained‐release behavior, with 80.42% total drug delivery. Through its matrix‐regulated delivery mechanism, the hydrogel achieved both prolonged release duration and enhanced therapeutic loading efficiency.

### 2.4 Photodynamic Synergistic Antibacterial Activity of Chiral Nanofibers

**Figure 5:**
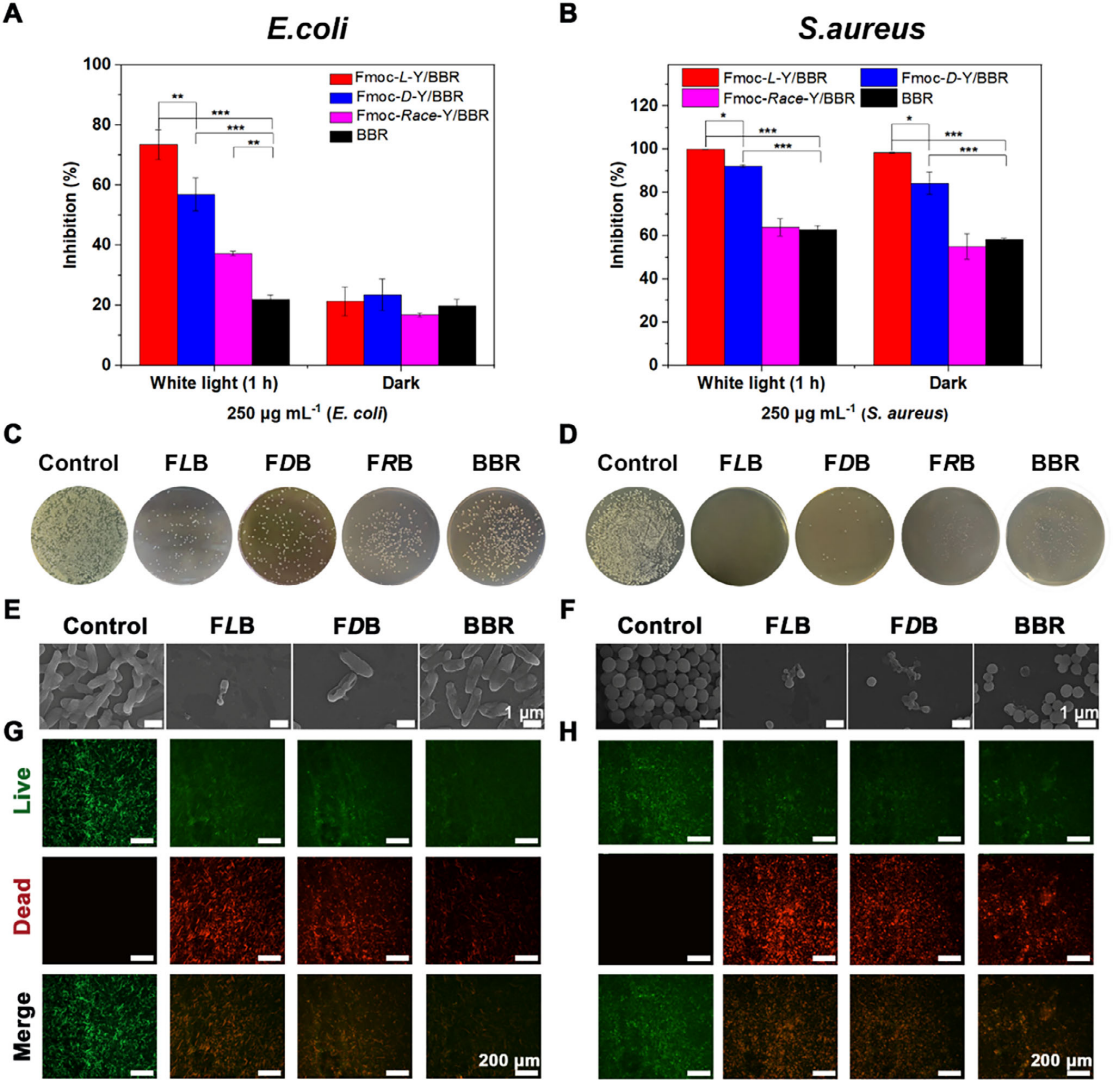
Photodynamic synergistic antibacterial properties of Fmoc‐Y/BBR nanofibers. A,B) Photodynamic synergistic antibacterial activity of *E. coli* and *S. aureus* treated with Fmoc‐Y/BBR chiral assemblies. C,D) Photographs of agar plate smears of *E. coli* and *S. aureus* treated with Fmoc‐Y/BBR chiral assemblies or BBR solution. Incubated under 20 mW cm^−2^ white light for 1 h. E,F) SEM images of *E. coli* and *S. aureus* bacterial cells treated with Fmoc‐Y/BBR chiral assemblies or BBR solution. Incubated under 20 mW cm^−2^ white light for 1 h. scale bars: 1 µm. G,H) Representative fluorescence diagram of live‐dead bacteria of *E. coli* and *S. aureus* treated with Fmoc‐Y/BBR chiral assemblies or BBR solution. Incubated under 20 mW cm^−2^ white light for 1 h. Scale bars: 200 µm. All samples were treated at a concentration of 250 µg mL^−1^, with BBR content at 8.6 µg mL^−1^. Values are expressed as the means ± SD, ^*^ *p* < 0.05, ^**^ *p* < 0.01, and ^***^ *p* < 0.001 (n = 3).

To systematically evaluate the antibacterial activity and photodynamic synergy of Fmoc‐Y/BBR chiral nanofibers, we conducted turbidimetric quantification (OD_600_ monitoring), plate spreading, streaking assays, and live‐dead bacteria staining experiments. Control experiments revealed that Fmoc‐Y self‐assemblies alone exhibited weak antibacterial activity (Figure S15, Supporting Information, inhibition rate <20%). To assess the role of chirality in antimicrobial efficacy, we compared enantiomeric co‐assemblies of Fmoc‐*L*‐Y/BBR and Fmoc‐*D*‐Y/BBR (250 µg mL^−1^ co‐assembly containing 8.6 µg∙mL^−1^ BBR) against *E. coli* (Gram‐negative, ATCC 25 922) and *S. aureus* (Gram‐positive, ATCC 6538P). Their antimicrobial performance was evaluated using minimum inhibitory concentration (MIC) data (Figure S16, Supporting Information). As shown in **Figure** **5**A, dark‐condition experiments indicated limited bactericidal efficacy against *E. coli* for both free BBR (control) and Fmoc‐Y/BBR co‐assembly, with inhibition rates of 21.3% (*L*‐form) and 23.5% (*D*‐form). Notably, white light irradiation (20 mW cm^−2^, 1 h) induced significant photodynamic enhancement, increasing inhibition rates to 74.5% (*L*‐form) and 56.9% (*D*‐form), demonstrating statistically superior performance by the *L*‐enantiomer (*p*<0.01).

Similar stereoselective antibacterial properties were observed against *S. aureus*. Under dark conditions, Fmoc‐*L*‐Y/BBR exhibited a 98% inhibition rate, significantly outperforming its *D*‐counterpart (82.3%, *p*<0.05). The racemic mixture showed ≈54.5% efficacy, comparable to free BBR. Photodynamic activation further enhanced antimicrobial outcomes, achieving complete inhibition (100%) for the *L*‐form and 92.2% for the *D*‐form, while the racemic mixture, free BBR, and control groups showed no significant change (Figure 5B). Control experiments with physical mixtures (Fmoc‐Y&BBR) confirmed the necessity of co‐assembly. The mixtures showed negligible activity against *E. coli* and significantly weaker inhibition against *S. aureus* compared to the co‐assembled nanofibers, underscoring the critical role of supramolecular chirality in enhancing photodynamic antibacterial efficacy (Figure S17, Supporting Information). Collectively, these findings demonstrate that chiroptical properties critically influence interactions between nanofibers and bacteria. *L*‐type nanofibers exhibit stereoselective antibacterial enhancement, while photodynamic activation achieves broad‐spectrum antimicrobial activity via photochemical mechanisms. The superior performance of Fmoc‐*L*‐Y/BBR assembly likely arises from their optimized supramolecular chirality, which matches bacterial membrane topology, thereby facilitating reactive oxygen species generation and targeted membrane disruption.

Following the initial experiments, we further validated the antimicrobial efficacy using standardized plate spreading and streaking assays (Figure 5C,D, Figure S18, Supporting Information). Under white light illumination, colony growth analysis showed that the Fmoc‐Y/BBR chiral assemblies exhibited significantly enhanced antimicrobial performance compared with control groups, thereby demonstrating superior photodynamic synergistic antibacterial effects. Notably, the Fmoc‐*L*‐Y/BBR formulation showed distinct advantages over its *D*‐enantiomer (Fmoc‐*D*‐Y/BBR) against both *S. aureus* and *E. coli* strains. Additionally, we used scanning electron microscopy (SEM) to analyze bacterial morphology (Figure 5E,F). Control groups displayed intact cellular structures with smooth membrane surfaces. In striking contrast, bacteria treated with Fmoc‐Y/BBR chiral assemblies exhibited severe cell wall collapse and cytoplasmic leakage. Importantly, the Fmoc‐*L*‐Y/BBR group induced more extensive structural damage compared with the *D*‐enantiomer assembly, with observable membrane fragmentation and complete cellular disintegration in many specimens.

We quantitatively assessed bacterial viability following photodynamic treatment using the BacLight Bacterial Survival Kit coupled with confocal laser scanning microscopy (CLSM). This dual‐fluorescence system enables clear differentiation between viable (green fluorescence) and membrane‐compromised (red fluorescence) cells. As shown in Figure 5G,H, control groups exhibited uniform green fluorescence across both *E. coli* and *S. aureus* populations. While the BBR treatment group showed mixed fluorescence signals indicating partial bactericidal effects, the Fmoc‐Y/BBR chiral assembly demonstrated dramatic increases in red fluorescence intensity, particularly against *S. aureus* where near‐complete erythrocyte staining was observed. Notably, the photodynamic antibacterial efficacy showed clear enantiomeric dependence. Fmoc‐*L*‐Y/BBR assembly achieved significantly higher bacterial mortality rates compared to their *D*‐configured counterparts. The observed enantiomeric performance differential strongly supported the existence of intrinsic chiral recognition mechanisms in microbial pathogens, potentially mediated through stereospecific interactions with bacterial membrane components (Figure 1C).

Bacterial biofilms present a major clinical challenge due to their dual defense mechanisms mediated by extracellular matrices against antimicrobial agents and host immunity.^[^ ^58^, ^59^, ^60^ ^]^ The 2,3‐Bis‐(2‐methoxy‐4‐nitro‐5‐sulfophenyl)‐2H‐tetrazolium‐5‐carboxanilide (XTT) assays demonstrated that the Fmoc‐*L*‐Y/BBR chiral assembly synergistically enhanced antibiofilm efficacy when combined with photodynamic therapy (Figure S20, Supporting Information). Under dark conditions, the assemblies showed baseline inhibition rates of 14.2% against *E. coli* biofilms and 38.8% against *S. aureus* biofilms; these rates significantly increased to 59.8% and 73.8% elimination rates, respectively, upon white light irradiation. Colorimetric analysis revealed distinct biofilm responses, control groups maintained intact coloration, free BBR showed negligible disruption, while the chiral assemblies induced visible color fading even in the dark. After 1 h of light exposure, *E. coli* biofilms turned pale yellow, and *S. aureus* biofilms developed an egg‐white‐like transparency. SEM analysis confirmed substantial matrix disintegration and reduced bacterial density in treated samples, in contrast to the densely packed aggregates observed in control groups. Collectively, these findings indicate that the chiral assemblies employ a chemo‐photodynamic synergistic mechanism to physically disrupt biofilm architecture while overcoming microbial resistance, establishing a dual‐action therapeutic strategy with potential for developing broad‐spectrum antimicrobials against recalcitrant biofilm‐associated infections.

### 2.5 Morphological and Mechanistic Insights into the Antibacterial Action of Chiral Nanofibers

**Figure 6:**
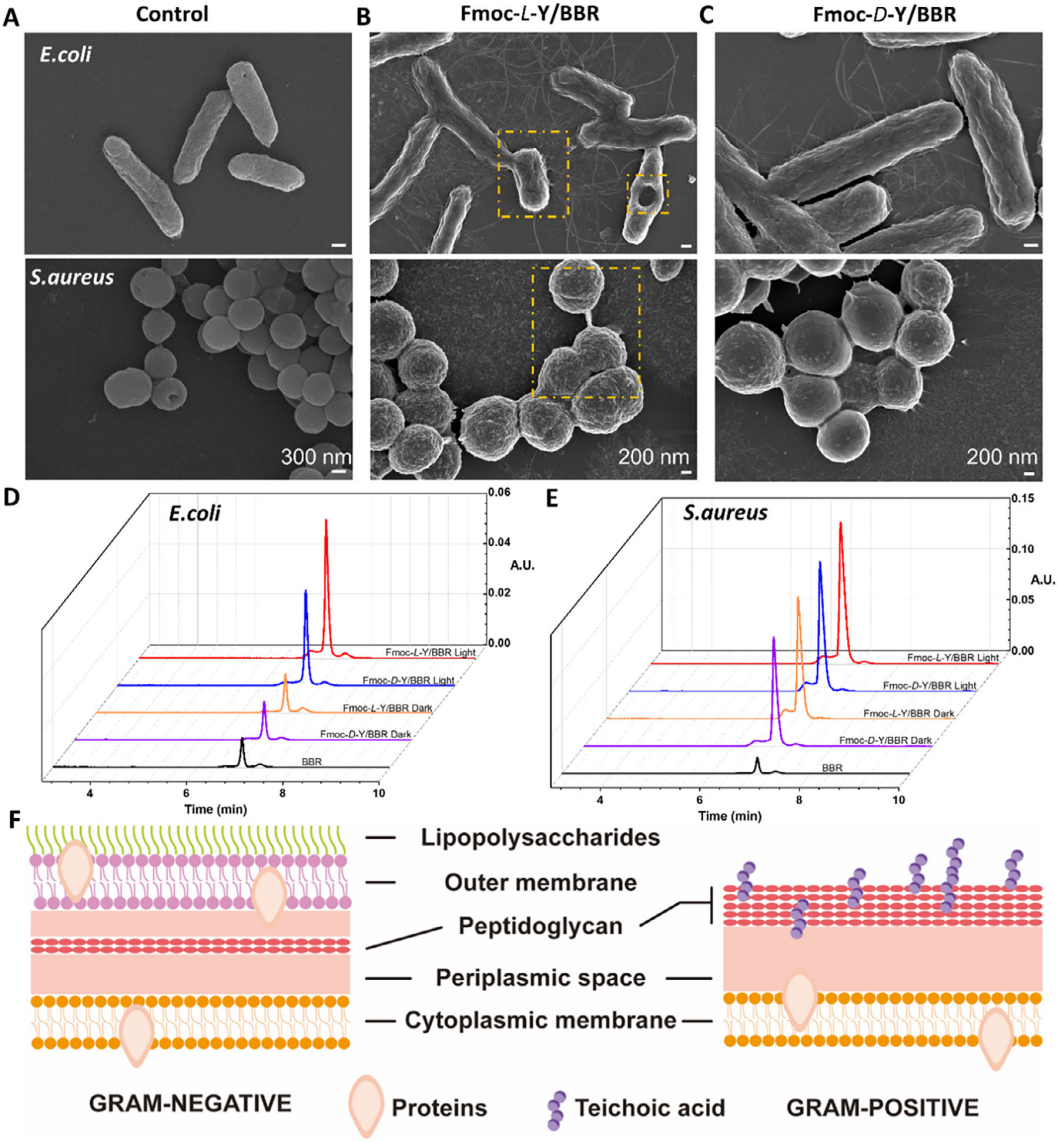
Photodynamic‐driven antibacterial mechanism of Fmoc‐Y/BBR chiral nanofibers. A) SEM images of normal cells of *E. coli* and *S. aureus* bacteria. Scale bars: 300 nm. B) SEM images of *E. coli* and *S. aureus* bacterial cells treated with Fmoc‐*L*‐Y/BBR chiral assembly. Incubated under 20 mW cm^−2^ white light for 1 h. Scale bars: 200 nm. C) SEM images of *E. coli* and *S. aureus* bacterial cells treated with Fmoc‐*D*‐Y/BBR chiral assembly. Incubated under 20 mW cm^−2^ white light for 1 h. Scale bars: 200 nm. Quantification of intracellular BBR in *E. Coli* D) and *S. aureus* E) under different treatments. The light groups were illuminated for 1 h under white light at an intensity of 20 mW cm^−^^2^. F) Schematic diagram of the structural components of the bacterial cell membranes of Gram‐negative & Gram‐positive bacteria.

Comparative SEM analyses revealed distinct morphological responses of *E. coli* and *S. aureus* to therapeutic interventions. The untreated control groups of both *E. coli* and *S. aureus* maintained their characteristic morphologies, rod‐shaped with smooth surfaces for *E. coli* and spherical with clustered coccal architecture for *S. aureus* (**Figure** **6**A). Following Fmoc‐*L*‐Y/BBR treatment, both bacterial species exhibited ultrastructural damage, including nano‐scale membrane invaginations, localized perforations, and cytoplasmic content extrusion in *E. coli*, along with significant surface roughness, cytoplasmic leakage, and adhesion in *S. aureus* (Figure 6B, Figure S21, Supporting Information). In contrast, Fmoc‐*D*‐Y/BBR treatment induced moderate morphological alterations, *E. coli* maintained an approximately rod‐shaped morphology but with distinct surface texturing, while *S. aureus* exhibited noticeable shape distortion with cytoplasmic leakage (Figure 6C).

To elucidate the antibacterial mechanism of the chiral assemblies, we systematically evaluated their effects on *E. coli* and *S. aureus* by monitoring membrane permeability using the 1‐N‐phenylnaphthylamine (NPN)9‐Fluorenylmethyloxycarbonyl‐tyrosine assay,^[^ ^61^ ^]^ followed by quantifying intracellular BBR accumulation. The NPN‐based membrane permeability assays (Figure S22, Supporting Iinformation) indicated that *P*‐helical nanofibers induced the most substantial increase in fluorescence under light exposure, outperforming both the dark‐control groups and the *M*‐helix counterparts. These results confirm that *P*‐helix nanofibers disrupt bacterial membranes more effectively, exhibiting chirality‐dependent and photo‐enhanced activity across species. Consistent with these findings, UPLC‐based quantification of intracellular BBR demonstrated a clear positive correlation with antibacterial activity.^[^ ^23^ ^]^ As summarized in Figure 6D,E, the *P*‐helix nanofibers promoted greater BBR uptake than the *M*‐helix analogs across all conditions, suggesting a chiral‐dependent uptake behavior. Notably, light irradiation further enhanced BBR accumulation in the *P*‐helix group, with this photo‐enhancement being more evident in *E. coli*, a phenomenon potentially linked to chiral‐selective interactions with bacterial membranes.

This differentiated antibacterial efficacy might arise from the interplay between bacterial envelope architecture and the stereochemical mechanism of antimicrobial agents (Figure 6D). Gram‐negative bacteria (e.g., *E. coli*) employ a multilayered defense system comprising an outer lipopolysaccharide membrane (7–8 nm) shielding a thin peptidoglycan layer (2–7 nm), which collectively restrict molecular penetration to the cytoplasmic membrane.^[^ ^17^ ^]^ In contrast, Gram‐positive bacteria (e.g., *S. aureus*) rely on a thick peptidoglycan matrix (20–80 nm) embedded with anionic teichoic acids, creating an electrostatic interface vulnerable to positively charged BBR interaction. This architectural distinction explains how the chiral nanofibers preferentially enhance pathogen adsorption through chirality‐match recognition in Gram‐positive bacteria, subsequently destabilizing the teichoic acid‐peptidoglycan supramolecular network through molecular interactions.

The observed stereoselectivity arises from precise geometric recognition at multiple biological interfaces. The right‐handed helical nanofibrils in Fmoc‐*L*‐Y/BBR complexes achieve three‐dimensional structural alignment with *L*‐phospholipid headgroups in Gram‐negative membranes, enabling a novel “drill‐bit” penetration mechanism. This chiral match allows sequential disruption of the lipopolysaccharide barrier followed by targeted delivery of BBR and ROS to compromise membrane integrity through synergistic action (Figure 1B). Conversely, the left‐handed Fmoc‐*D*‐Y/BBR nanofibers exhibit chiral mismatch with native lipid configurations, resulting in reduced membrane adhesion efficiency and ineffective payload release, where the outer membrane's structural hierarchy amplifies this stereochemical exclusion effect. This structure‐mechanism correlation highlights how bacterial envelope complexity governs nanotherapeutic efficacy through molecular‐scale geometric recognition.

### 2.6 In Vivo Application of Chiral Hydrogels for Healing Infected Wounds

**Figure 7:**
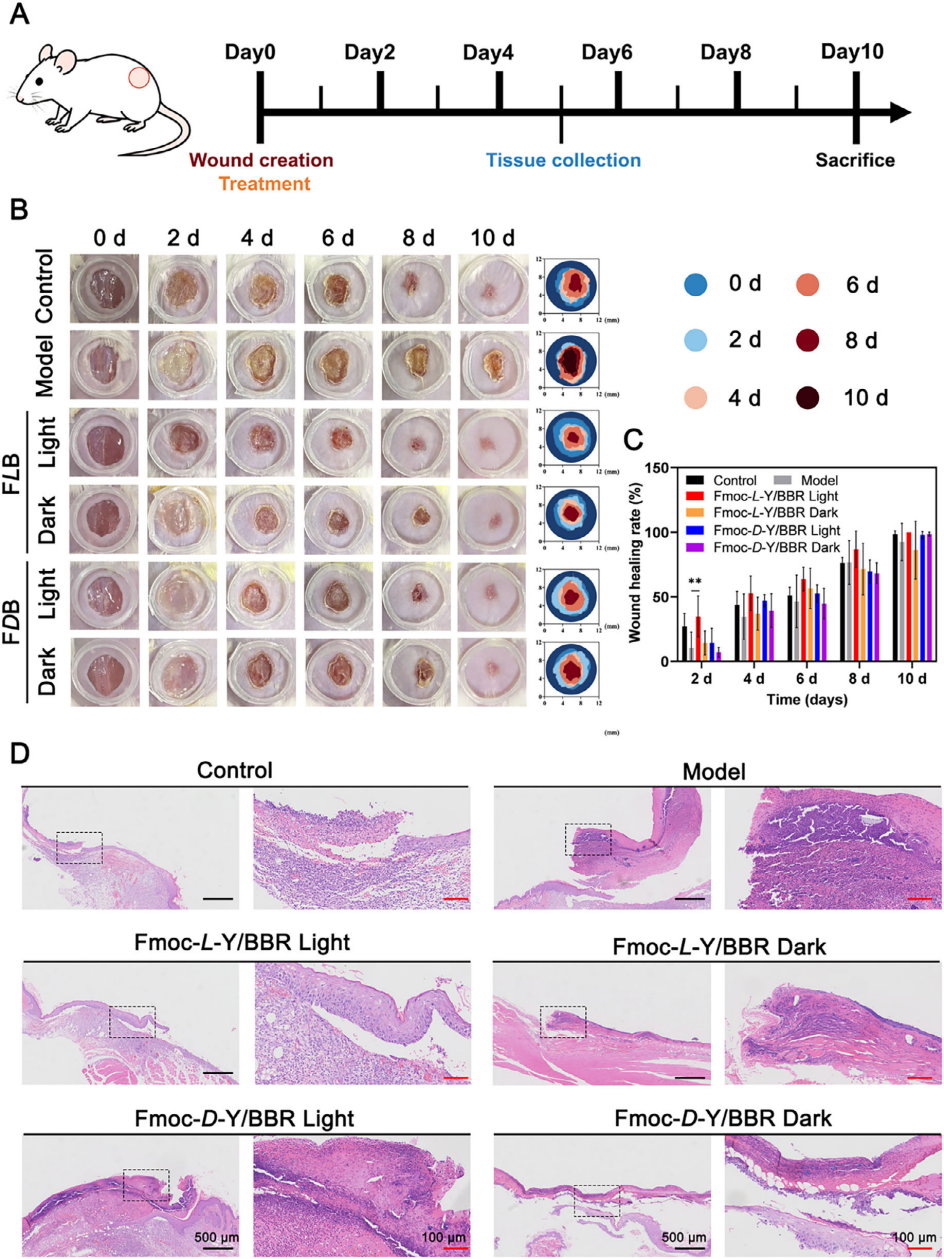
Effects of the Fmoc‐Y/BBR chiral hydrogels in promoting wound healing. A) Schematic diagram of wound healing in BALB/c mice. B) Representative photos of different groups of skin wounds on days 0, 2, 4, 6, 8 and 10. C) Wound healing area of PBS group, *S. aureus* + PBS group and *S. aureus* + Fmoc‐Y/BBR chiral hydrogels groups with or without white light at different time points. The light groups were illuminated for 0.5 h under white light at an intensity of 20 mW cm^−^^2^. Values were expressed as the means ± SD. ^**^ *p* < 0.01 (n = 5). D) Representative pictures of H&E‐stained histological sections on days 6 after different treatments.

To evaluate the photodynamic antibacterial activity and wound‐healing efficacy of the chiral hydrogel in vivo, we established a mouse model of *S. aureus*–infected skin wounds. As illustrated in the experimental timeline (**Figure** **7**A), hydrogels were topically applied to the wounds followed by corresponding light exposure, with wound healing monitored at predetermined intervals. Macroscopic examination after two days of treatment revealed distinct differences among the groups. The Model group exhibited severe infection accompanied by pus formation, whereas wounds treated with Fmoc‐*L*‐Y/BBR under white light (Fmoc‐*L*‐Y/BBR Light) remained clean without signs of bacterial growth. This group also demonstrated the most rapid wound contraction and scab formation (Figure 7B). By day 8, the wound healing rate in the Fmoc‐*L*‐Y/BBR Light group exceeded 85%, significantly higher than that in all other groups (Figure 7C). Enhanced formation of new granulation tissue in this group was further emphasized in wound simulation images.

Histological analysis (H&E staining) on day 5 corroborated these observations. Both the Fmoc‐*L*‐Y/BBR Light group and the positive control group achieved nearly complete re‐epithelialization, showing well‐defined neo‐dermis and epidermis along with markedly reduced inflammatory cell infiltration (Figure 7D). In contrast, groups that did not receive light exposure (Dark) and the Model group displayed minimal new epidermal formation and sustained substantial presence of inflammatory cells. Together, these results indicate that the Fmoc‐Y/BBR chiral hydrogel, upon activation by white light, effectively eliminates wound bacteria through a potent photodynamic antibacterial mechanism. This action establishes a favorable microenvironment for tissue regeneration, leading to significantly accelerated healing of infected wounds.

## 3 Conclusion

In summary, we demonstrate that supramolecular chirality plays a critical role in enhancing the photodynamic antibacterial efficacy of co‐assembled nanostructures derived from natural herbal components. By co‐assembling the achiral alkaloid BBR with chiral Fmoc‐tyrosine enantiomers, we constructed helical nanofibers with tunable handedness. This specific supramolecular arrangement significantly amplified the photodynamic activity, leading to efficient, light‐triggered ROS generation. The antibacterial mechanism was systematically investigated. By employing assays to quantify intracellular BBR and to monitor membrane permeability via NPN uptake, complemented by SEM observation, we found that the right‐handed nanofibers accumulated more effectively in *E. coli* and *S. aureus* and caused greater membrane disruption than their left‐handed counterparts. This stereoselective, chirality‐matched interaction underpinned their markedly superior photodynamic bactericidal activity. These in vitro findings were strongly supported by in vivo experiments using a mouse model of *S. aureus*‐infected wound healing. Treatment with the right‐handed nanofibers under white light illumination led to effective bacterial clearance, reduced inflammation, and significantly accelerated wound closure and tissue regeneration, outperforming other control groups.

Collectively, our work demonstrates how hierarchical chirality, spanning from the molecular to the supramolecular scale, can direct biological interactions, creating a bridge between traditional herbal medicine and contemporary nanotherapy. It pioneers a strategy for constructing highly efficient antimicrobial systems by strategically organizing inherent pharmacophores of natural products, such as BBR, into controlled chiral nanostructures. This methodology, which synergistically combines precise nanostructure design, photodynamic activation, and chirality‐based targeting, presents a potent approach to address antibiotic‐resistant infections and advance the application of bioactive natural products.

## 4 Experimental Section

### Materials

BBR used in this experiment was purchased from Sigma–Aldrich, Shanghai, China, with a purity of more than 95%. Fmoc‐Y and sodium hydroxide were purchased from Shanghai Jizi Biochemical Technology Co. The •OH detection kit was from Nanjing Jianjian Bioengineering Institute (A018‐1‐1, China). *S. aureus* (ATCC 6538P) and *E. coli* (ATCC 25 922) strains were cultured in this study. XTT sodium salt was purchased from Shanghai McLean Biochemical Technology Co.

### General Procedure for the Preparation of the Nanofibers

Preparation of Fmoc‐Y nanofibers:Fmoc‐*L*‐Y or Fmoc‐*D*‐Y powder (14.00 mg) was dissolved in 0.5 mL of phosphate‐buffered saline (PBS) and the pH was adjusted to 8 with NaOH aqueous solution, then ultrasonicated for 10 min at room temperature. The suspension was subsequently heated at 85 °C until phase stratification occurred. Following this, 0.5 mL of deionized water was introduced, and the mixture was incubated at room temperature for 2 h to form the final Fmoc‐Y hydrogels composed of nanofibers. Preparation of Fmoc‐Y/BBR nanofibers: Initially, 14.00 mg of Fmoc‐*L*‐Y or Fmoc‐*D*‐Y powder was dissolved in 0.5 mL of alkaline PBS (PH = 8.0), ultrasonicated for 10 min, and heated at 85 °C until stratification. 0.5 mL of 1 mg mL^−1^ BBR aqueous solution was added to the above clarified solution. The mixture was then allowed to stand at room temperature for 2 h to form Fmoc‐*L*‐Y/BBR or Fmoc‐*D*‐Y/BBR hydrogels composed of chiral nanofibers. For the racemic variant (Fmoc‐*Rac*‐Y/BBR), equal amounts of Fmoc‐*L*‐Y and Fmoc‐*D*‐Y powders (7.00 mg each) were processed under the same conditions, with BBR solution incorporated at the gelation stage.

### Measurement of Hydroxyl Radical Generating Capacity

 •OH generating capacity was determined using the •OH assay kit. Prepared blank tubes, control tubes, and 4 sets of test tubes, added double‐distilled water and hydrogen peroxide (H_2_O_2_) to the blank tube and control tube, respectively. Fmoc‐Y/BBR samples with or without white light irradiation were incubated with the reagent application solution for 10 min at r.t. Griess reagent was then added to develop the color for 20 min. Absorbance was measured at the wavelength of 550 nm. The ability to generate •OH was calculated as:

•OH generating capacity

(1) $$\begin{array}{ccc} \left(U\;mL-1\right) & = & \left(Ameasure\;-\;Acontrol\right)\;/\;\left(Astandard\;-\;Ablank\right)\\ & & \times \;Cstandard\;\times \;1/Vsample\;\times \;N \end{array}$$

The standard was 0.03% H_2_O_2_ solution. C_standard_: standard solution, 8.824 mmol L^−1^; V_sample_: sampling volume, 0.1 mL; N: dilution of samples before testing.

### HaCat Cytotoxicity Assay

HaCat cells were resuscitated and cultured, 96‐well plates were laid out at 5000 cells per well when the cells entered the logarithmic growth phase. The 96‐well plates were placed in a thermostatic incubator for 24 h. Fmoc‐*L*‐Y/BBR and Fmoc‐*D*‐Y/BBR assemblies were diluted with complete cell culture medium and added to the 96‐well plates to achieve the final concentrations of 50, 100, 150 and 200 µg mL^−1^, incubated for 24 h. The absorbance of the samples was detected at 490 nm using MTT staining method. The inhibitory rate was calculated in the following Equation (2):

(2) $$\begin{array}{ccc} inhibition\;\% & = & \left[1-\left(ODSample\;group\;-\;ODBlank\;group\right)\right.\\ & & \left./\left(ODControl\;group-ODBlank\;group\right)\right]\;\times \;100\% \end{array}$$

### Antibacterial Assays

In this study, Gram‐positive *S. aureus* (ATCC 6538P) and Gram‐negative *E. coli* (ATCC 25 922) were used to evaluate the in vitro bacterial inhibitory activities of Fmoc‐Y/BBR. Fmoc‐Y/BBR and BBR were diluted with medium to the appropriate concentration in 48‐well plates, making the final volume of each well 500 µL, then 30 µL of *S. aureus* and *E. coli* (2 × 10^6^ CFU mL^−1^) were added to each well. For the light‐induced toxicity assay, the 48‐well plates were exposed to white light for 1 h, and the control group was placed in the dark, followed by incubation of the 48‐well plates in a constant temperature incubator at 37 °C for 12 h. Bacterial growth was observed using an enzyme marker at an optical density of 600 nm. The diluted bacterial suspension after incubation was inoculated onto nutrient agar by smearing and scribing method, then incubated in a constant temperature incubator at 37 °C for 14 h to verify the bacterial growth. After incubation, 48‐well plates were added with LIVE/DEAD BacLight^TM^ bacterial activity kit for staining after administration and rinsing, and photographed in the dark with a fluorescent inverted microscope.

### Morphological Observation of Bacteria

As described above, *S. aureus* and *E. coli* were co‐cultured with 250 µg mL^−1^ of Fmoc‐*L*‐Y/BBR, Fmoc‐*D*‐Y/BBR and the corresponding concentration of BBR for 12 h, respectively. Bacteria were obtained by centrifugation (3000 rpm, 10 min) and then washed three times with PBS. Samples were fixed with 2.5% glutaraldehyde at 4 °C for 4 h and dehydrated by gradient increase of ethanol concentration (30, 50, 70, 80, 90, 95 and 100%) for 10 min each. Finally, SEM was used to observe the microscopic morphology of bacteria.

### Bacterial Biofilm Removal Effect Assay

To test the bacterial biofilm inhibition ability, 200 µL of *S. aureus* and *E. coli* was incubated in 96‐well plates for 24 h at 37 °C, after which the medium was removed to add 250 µg mL^−1^ of Fmoc‐*L*‐Y/BBR, Fmoc‐*D*‐Y/BBR and an equal amount of BBR for co‐incubation. The medium was removed after 24 h of incubation, and then the incubation was continued for 2 h by adding XTT staining solution, and the absorbance was measured at 450 nm. Biofilms were incubated on sterilised silicon wafers placed in 12‐well plates and after 24 h of administration, they were washed with PBS and fixed with 2.5% glutaraldehyde for 4 h. The growth of bacterial biofilm was observed by SEM.

### In Vitro Drug Release Experiment

A mixture of 18 mL was prepared as a drug release medium by aspirating 2 mL of the sample into the dialysis bag and mixing deionized water and PBS solution of pH 7.4 in a 1:1 ratio. At the same time, BBR solution of the same concentration was prepared as a control group. The dialysis bag was immersed in the release medium and stirred at 37 °C and 300 rpm. At preset time points (0.5, 1, 2, 4, 6, 8, 12 and 24 h), 100 µL of sample solution was aspirated from the drug release medium for determination. The entire experimental procedure was repeated three times.

### NPN Assay for Assessing Bacterial Membrane Permeability

NPN is a lipophilic dye exhibiting weak fluorescence in aqueous solutions but intense fluorescence in hydrophobic media. When bacterial membranes were damaged, their permeability alters, allowing NPN to migrate into the bacteria where it emits fluorescence. First, centrifuged the *S. aureus* suspension at 8000 rpm for 5 min. Discarded the supernatant and resuspended the bacteria in PBS buffer to a concentration of 1 × 10^8^ CFU mL^−1^. Subsequently, added 50 µL of NPN solution (40 µm) and 50 µL of bacterial suspension to each well of a 96‐well plate. Concurrently, added 100 µL 1 mg mL^−1^ (4 × MIC) of Fmoc‐*L*‐Y/BBR and Fmoc‐*D*‐Y/BBR to each well, with three wells receiving 100 µL of PBS as control. Fluorescence signals were monitored using a multifunctional microplate reader (excitation wavelength λ_ex_ = 350 nm, emission wavelength λ_em_ = 420 nm).

### Detection of BBR Aggregation within S. aureus via UPLC

1 mg mL^−1^ of BBR and Fmoc‐Y/BBR were co‐cultured with *S. aureus* at 37 °C for 6 h. Subsequently, the bacteria were harvested by centrifugation at 3000 rpm for 5 min. Following three washes with PBS buffer, the bacterial suspension was subjected to ultrasonication in methanol for 2 h and filtered through a 0.22 µm filter. The final BBR content in the bacteria was determined using Waters company's UPLC instrument.

### In Vivo Wound Healing Experiment of S. aureus‐Infected Skin in Mice

48 adult male BALB/c mice (6–8 weeks old) used in this experiment were procured from SPF (Beijing) Biotechnology Co., Ltd. The experimental protocol was approved by the Animal Research Ethics Committee of Beijing University of Chinese Medicine (Approval No.: BUCM‐BUCM20250908‐001) and conducted in strict compliance with ethical guidelines for animal research.

Mice were acclimatised for one week prior to modelling. During the experiment, after anaesthesia with 1% sodium pentobarbital, an 8 mm circular incision was made in the dorsal skin. Animals were randomly assigned to four groups: 1) PBS control group. 2) *S. aureus* infection group. 3) *S. aureus* infection + Fmoc‐*L*‐Y/BBR hydrogel under light irradiation group. 4) *S. aureus* infection + Fmoc‐*L*‐Y/BBR hydrogel under dark group. 5) *S. aureus* infection + Fmoc‐*D*‐Y/BBR hydrogel under light irradiation group. 6) *S. aureus* infection + Fmoc‐*D*‐Y/BBR hydrogel under dark group. *S. aureus* infection was inoculated with 50 µL of 2 × 10^6^ CFU mL^−1^ bacterial suspension. In *S. aureus* infection‐related groups, a 30 min *S. aureus* infection was performed immediately after incision creation, followed by treatment with either PBS or hydrogels. In the hydrogel treatment groups, mice in the light‐exposure subgroup were incubated under 20 mW cm^−2^ white light for 30 min following hydrogel administration. Wound images were captured at days 0, 2, 4, 6, 8 and 10, with healing rates calculated by measuring wound area. These mice were randomly sacrificed from each group on days 5 and their internal organs and skin tissues were collected for subsequent experiments.

### Statistical Analysis

Statistical analyses were performed using SPSS version 20.0 (IBM, USA). The results of the experimental data were expressed as mean ± standard deviation. Independent samples *t*‐tests was used to compare statistical differences between groups. ^*^ *p* < 0.05, ^**^ *p* < 0.01 and ^***^ *p* < 0.001 were considered statistically significant.

## Conflict of Interest

The authors declare that they have no conflict of interest.

## Author Contributions

H.Z. and J.J. contributed equally to this work. H.Z., G.O., and M.L. conceived the study and designed the experiments, and H.Z. and J.J. led data curation and analysis with support from Z.W., C.S., and X.H. for experimental validation. J.J., X.H., and Y.L. contributed to the investigation of the antibacterial mechanisms and the animal experiments, while H.L., G.O., and M.L. provided the resources. Z.W., C.S., X.H., H.Y., and H.L. contributed to the scientific discussion. H.Z. and J.J. drafted the manuscript, which was critically revised by G.O. and M.L., and all authors discussed the results and commented on the manuscript.

## Supporting information



Supporting Information

## Data Availability

The data that support the findings of this study are available in the supplementary material of this article.
