# Supplementary material for: Amino Acid Directed Helical Assemblies of Natural Berberine for Chirality‐Dependent Photodynamic Antibacterial Therapy
Source: Adv Sci (Weinh). 2025 Nov 19;13(3):e14096. doi: 10.1002/advs.202514096 (PMC12806354; doi:10.1002/advs.202514096)
Supplement: Supplementary file 1 — Supporting Information [file ADVS-13-e14096-s001.pdf]

Supporting Information for

Amino Acid Directed Helical Assemblies of Natural Berberine for Chirality-Dependent Photodynamic Antibacterial Therapy

Haoqiang Zhao, Jingyi Jiao, Zhixia Wang, Chengyu Shi, Xinran Huang, Yixuan Lin, Haolin Yang, Haimin Lei,* Guanghui Ouyang,* and Minghua Liu**

H. Zhao, J. Jiao, Z. Wang, C. Shi, X. Huang, Y. Lin, H. Yang, H. Lei
School of Chinese Pharmacy
Beijing University of Chinese Medicine
Beijing 102488, China
E-mail: hq-zhao@bucm.edu.cn; hm_lei@126.com

H. Zhao, G. Ouyang, M. Liu
Beijing National Laboratory of Molecular Sciences and CAS Key Laboratory of Colloid Interface and Thermodynamics
Institute of Chemistry Chinese Academy of Sciences
ZhongGuanCun North First Street 2
Beijing 100190, China
E-mail: ouyanggh@iccas.ac.cn; liumh@iccas.ac.cn

H. Zhao and J. Jiao contributed equally to this work.

1. Supplementary Text

1.1 Scanning Electron Microscopy (SEM) test

SEM images were taken on S-4800 and SU8600 scanning electron microscopes with the accelerating voltage of 10 kV and the emission current of 10 μA.

1.2 Atomic force microscopic (AFM) test

AFM study was performed using Dimension FastScan (Bruker). Images were obtained in ScanAsyst mode under ambient conditions using the FastScan B probes. A small amount of wet hydrogels were placed on the freshly cracked mica plate for AFM studies. The materials were allowed to evaporate slowly in air and then vacuum dried for two days at room temperature.

1.3 Circular dichroism (CD) spectroscopy test

The CD spectra of BBR, Fmoc-Y and Fmoc-Y/BBR hydrogels were measured using Chirascan V100 (Applied Photophysics, UK) for the range 200–600 nm.

1.4 Circularly polarized luminescence (CPL) test

The CPL spectra of Fmoc-Y/BBR hydrogels were measured using a CPL-200 spectrophotometer (JASCO) with an excitation wavelength of 395 nm, covering the range of 400-800 nm. The acquired data was then analyzed utilizing JASCO's SpectraManager software.

1.5 Fluorescence spectra test

The BBR, Fmoc-Y and Fmoc-Y/BBR hydrogels were injected into a 0.1 cm quartz cuvette, and the fluorescence spectra were recorded using a Hitachi F-4500 Fluorescence Spectrophotometer at a voltage of 400 V and with a slit width of 5 nm. An excitation wavelength of 271 nm and 395 nm were employed. The fluorescence quantum yields were measured by the FluoroMax+ (HORIBA) spectrofluorometer.

1.6 Fluorescence lifetime test

Fluorescence lifetime measurements were recorded on the same spectrometer by using time-correlated single photon counting (TCSPC) at Edinburg FLS-980 fluorescence spectrometer.

1.7 UV-*vis* spectroscopy test

The absorbance of BBR, Fmoc-Y and Fmoc-Y/BBR assemblies at 200-600 nm were measured by HITACHI UH5300 spectrometer (Japan) after the samples was diluted with PBS/water = 1/1.

1.8 Infrared spectroscopy test

The FT-IR spectra of BBR, Fmoc-Y and Fmoc-Y/BBR assemblies were recorded in the frequency range of 400-4000 cm^{-1} with NicoletiS10, (Thermo, USA).

1.9 X-Ray Powder Diffraction test

Fmoc-*L*-Y, Fmoc-*L*-Y/BBR assemblies lyophilized powders and free BBR were subjected by XRD at 40 kV, 40 mA on a Rigku Ultima IV diffractometer with Cu-Kα radiation at room temperature with a 2θ range of 5°-30°.

1.10 ^{1}H-NMR spectroscopy test

Equimolar BBR, Fmoc-*L*-Y, and Fmoc-*L*-Y/BBR were dissolved in DMSO-d_6. ^{1}H-NMR spectra were recorded on an Avance IIIHD 400 MHz spectrometer (Bruker, America) with tetramethylsilane as an internal standard.

1.11 ESR spectroscopy test

Samples were exposed to white light and dark conditions and ESR measurements were performed on a JEOL JES FA200 spectrometer using a xenon lamp (50 mW·cm^{-2}) as the visible light source. 5,5-Dimethyl-1-pyrrolidine (DMPO) and 2,2,6,6-4-methyl-4-piperidone hydrochloride (4-oxo-TEMP) were used as specific scavengers of hydroxyl radicals (•OH) and singlet oxygen ($^{1}O_2$), respectively.

1.12 Zeta potential test

The zeta potential was determined using the dynamic light scattering (DLS) method with Malvern Zetasizer Nano-ZS-90 (Malvern Instruments, UK).

1.13 Haemolysis test

Water was used as a positive control and PBS as a negative control. The samples were mixed with erythrocytes to obtain a 4% erythrocyte solution. the absorbance at 570 nm was measured after centrifugation after incubation for 1 h at 37 ℃. The RHR (%) of each sample was calculated according to the following formula.

RHR (%) = $(OD_{example} - OD_{salt}) / (OD_{water} - OD_{salt}) \times 100\%$

Samples were considered haemocompatible when the RHR (%) of the sample was less than the internationally recognised 5%.

1.14 Stability test

The sample was left to stand at room temperature to observe whether it exhibited a certain degree of stability over time. 14.5 mg of Fmoc-Y/BBR lyophilised powder was weighed and added to 0.5 mL each of PBS and deionised water to observe whether it exhibited a sponge effect. In the experiment, the sample was destroyed or heated and then cooled, and after waiting for a period of time, it was observed whether it recovered.

1.15 Rheology test

All rheological measurements were performed using a rotary rheometer (DHR-2Rheometer, TA, USA). Dynamic frequency scanning were performed at 0.1% strain at frequencies from 0.01 to 100 Hz. The strain-dependent oscillation characteristics were measured at a frequency

of 1 Hz for shear strain variations ranging from 0.1% to 100%. During temperature scanning, the temperature range was set to 25–80 °C. The step strain rheological experiment was conducted in two steps: the first step involved testing the gel at a strain of 0.1%; the second step involves gradually increasing the strain from 0.1% to 100% and maintaining the gel at 100% strain for 50 seconds until complete failure. The entire testing process takes 700 seconds.

2. Supplementary Figures S1-S22

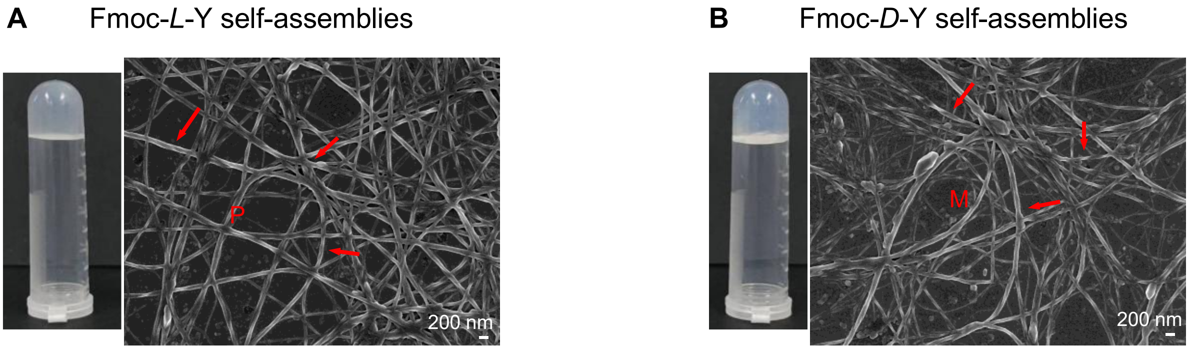

Figure S1. Sample vial photographs and SEM characterizations of Fmoc-Y self-assemblies. (A) Fmoc-*L*-Y; (B) Fmoc-*D*-Y. The sample was measured at a 50-fold dilution of hydrogel.

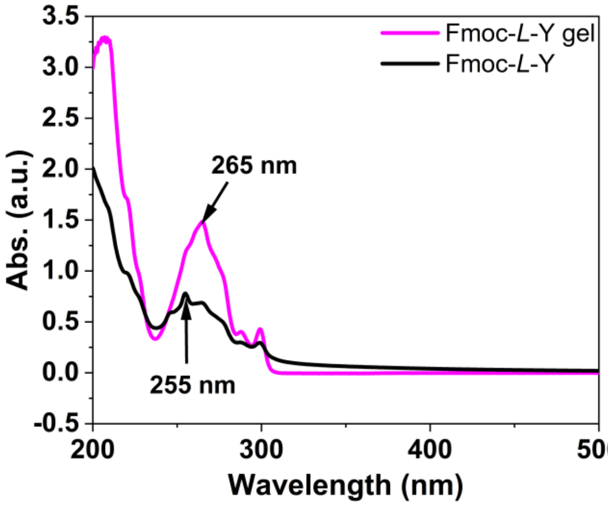

Figure S2. UV-*vis* spectra. Fmoc-*L*-Y hot solution (dark line, [Fmoc-*L*-Y] = 0.05 mM in PBS) and Fmoc-*L*-Y assemblies (pink line, [Fmoc-*L*-Y] = 0.1 mM in PBS).

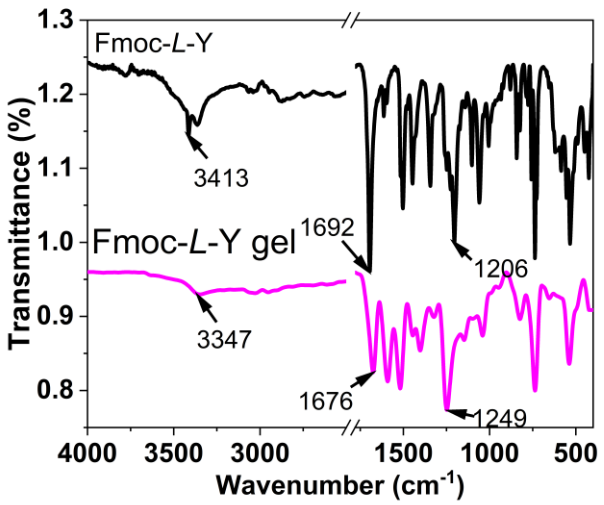

Figure S3. FT-IR spectra. Fmoc-*L*-Y powder (dark line) and lyophilized powder of Fmoc-*L*-Y assemblies (pink line).

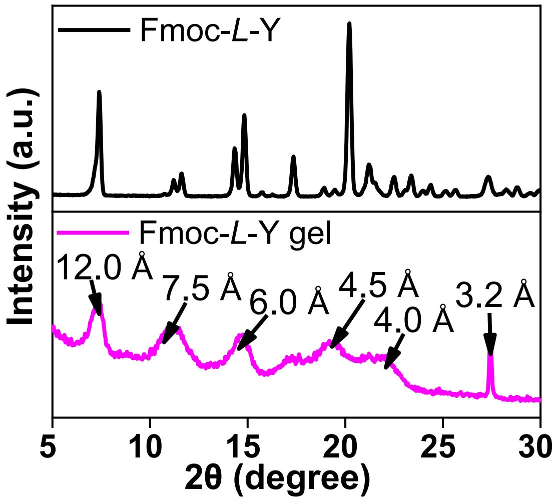

Figure S4. XRD patterns. Fmoc-*L*-Y powder (dark line) and lyophilized powder of Fmoc-*L*-Y assemblies (pink line).

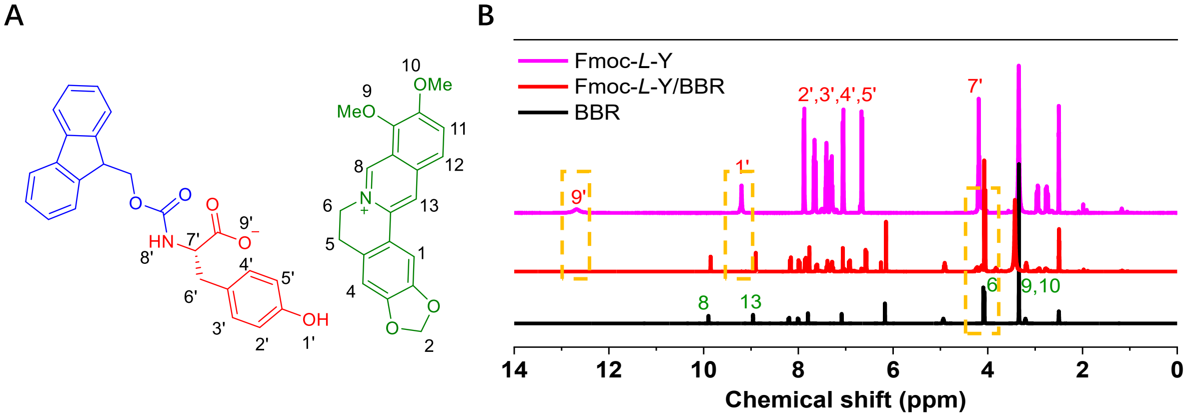

Figure S5. Molecular structures and stacked ^{1}H-NMR spectra. (A) The molecular structures of Fmoc-*L*-Y and BBR; (B) The stacked ^{1}H-NMR spectra of Fmoc-*L*-Y, BBR and Fmoc-*L*-Y/BBR (400 MHz, DMSO-d_6). [BBR] = [Fmoc-*L*-Y] = 24.8 mM.

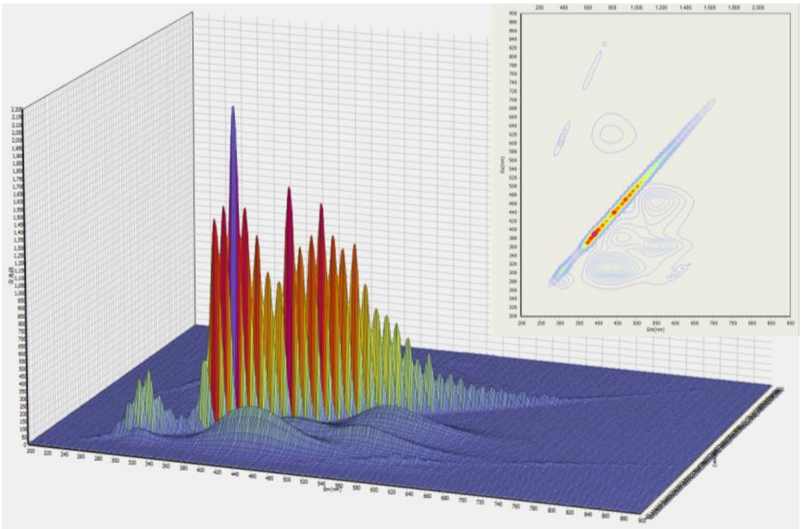

Figure S6. Three-dimensional fluorescence spectroscopy of Fmoc-*L*-Y/BBR co-assemblies.

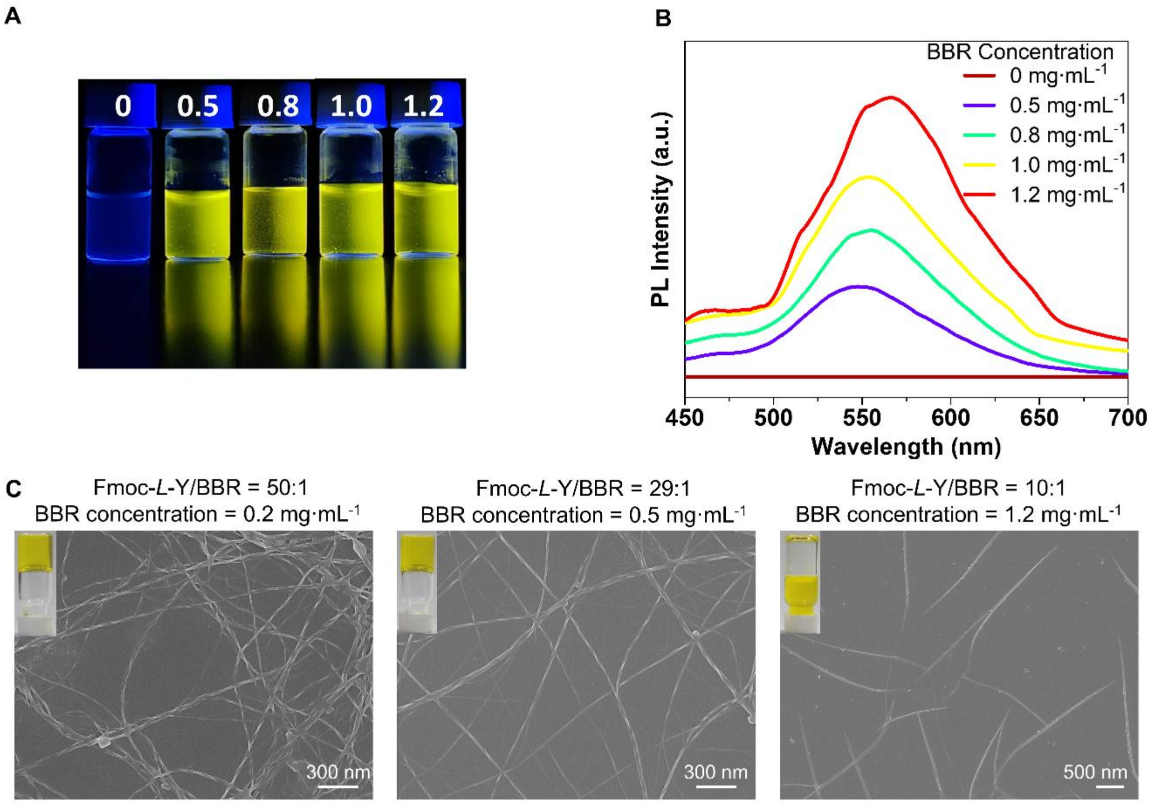

Figure S7. Digital photographs and fluorescence spectra of Fmoc-*L*-Y/BBR assemblies. (A) Fmoc-*L*-Y/BBR with different concentrations of BBR under 365 nm UV light; (B) Fluorescence spectra of Fmoc-*L*-Y/BBR with different concentrations of BBR. λ_{ex} = 390 nm. [Fmoc-Y] = 34.7 mM in PBS. (C) Molar ratio-dependent morphology of Fmoc-*L*-Y/BBR: Macroscopic hydrogel inversion and SEM images.

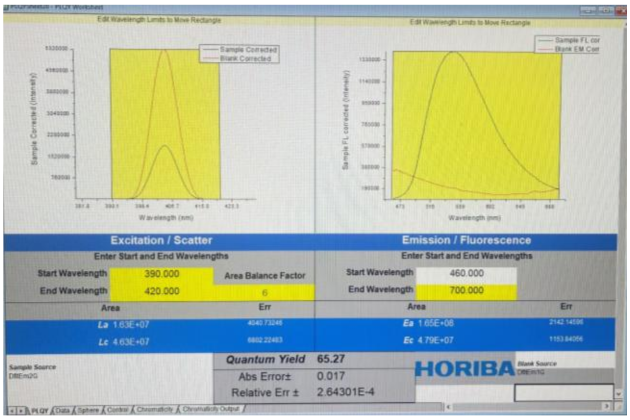

Figure S8. Fluorescence quantum yield of Fmoc-*L*-Y/BBR co-assemblies.

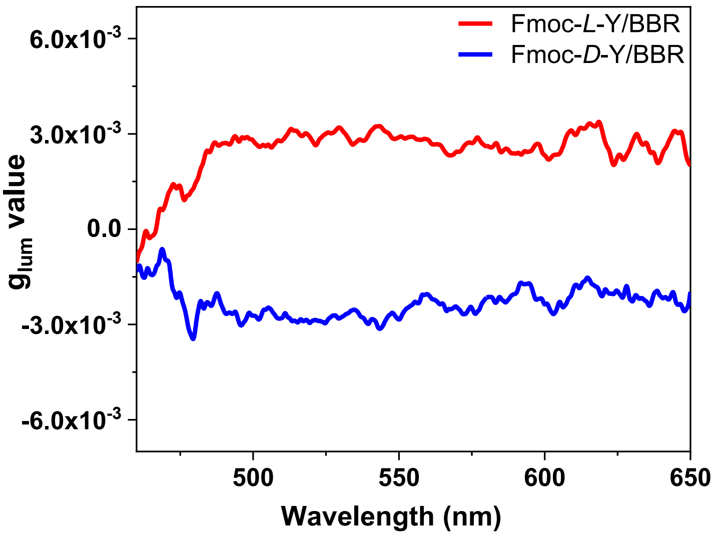

Figure S9. glum values of Fmoc-*L*-Y/BBR (red line) and Fmoc-*D*-Y/BBR (blue line) nanofibers.

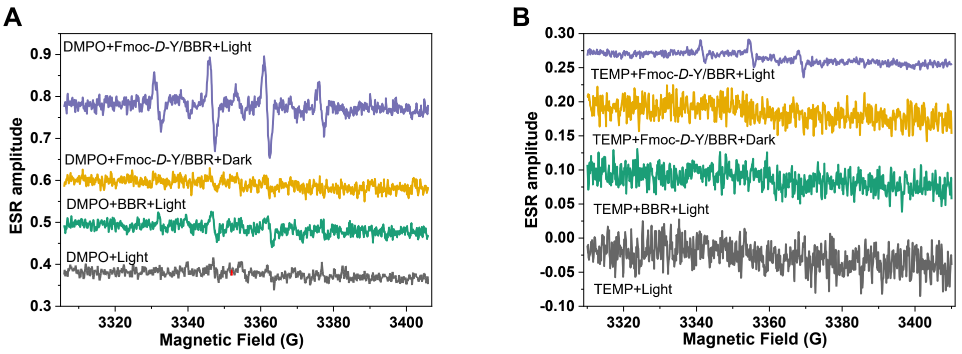

Figure S10. ESR spectra of Fmoc-*D*-Y/BBR co-assemblies. (A) DMPO ESR signals of •OH. (B) 4-oxo-TEMP ESR signals of $^1O_2$.

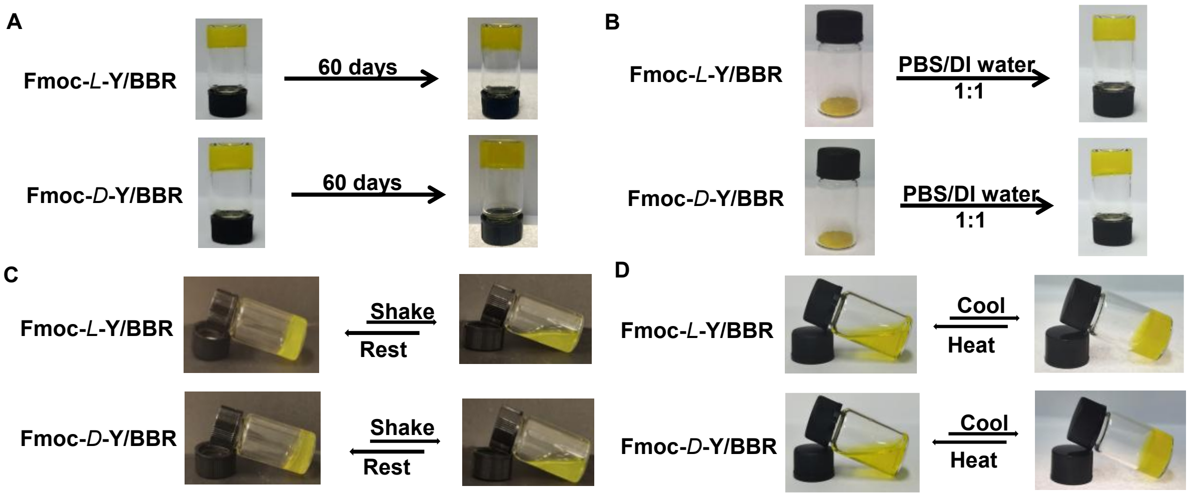

Figure S11. Digital photographs of Fmoc-Y/BBR chiral hydrogels. (A) the stability and (B) sponge effect verification experiment of Fmoc-Y/BBR system (14.5 mg·mL^{-1} freeze-dried powder redissolved); Gel-sol transitions of Fmoc-Y/BBR chiral hydrogels (C) shake & rest and (D) heat & cool.

Stability tests results showed that the Fmoc-Y/BBR chiral hydrogels could remain stable for at least 60 days when left stationary at room temperature. Additionally, they possessed a unique spongy effect, these lyophilized powders could gradually resume the hydrogels form when PBS/DI water was added. The chiral hydrogels also demonstrated good shear recovery and thermoreversibility.

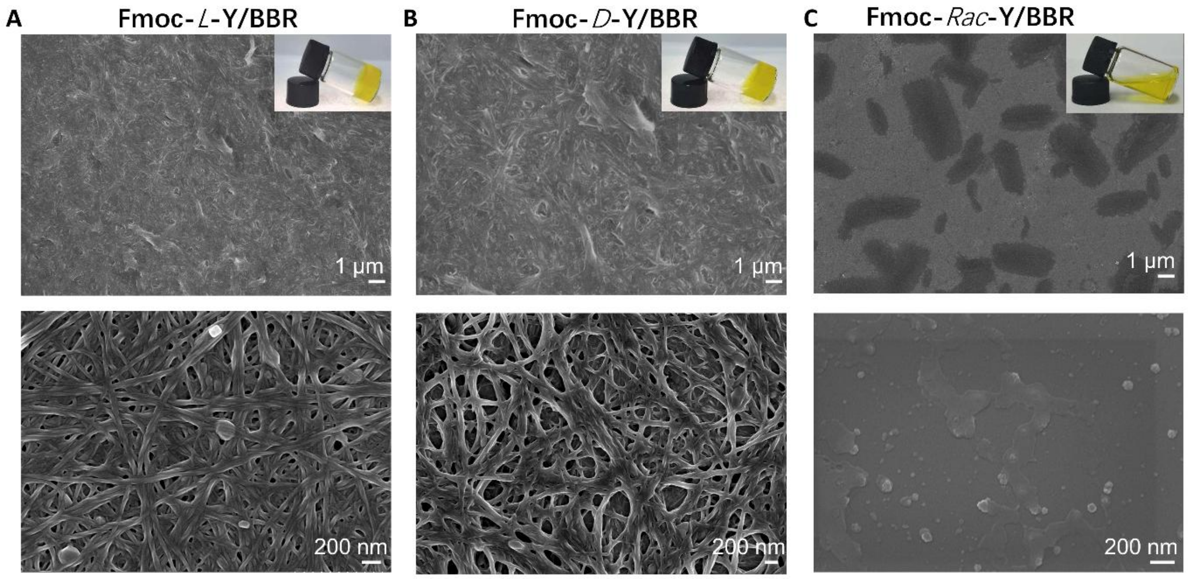

Figure S12. Sample vial photographs and SEM characterizations of Fmoc-Y/BBR assemblies. (A) Fmoc-*L*-Y/BBR; (B) Fmoc-*D*-Y/BBR; (C) Fmoc-*Rac*-Y/BBR. [Fmoc-Y] = 34.7 mM in PBS, Fmoc-Y/BBR = 29:1 in molar ratio. Scale bars: 1 μm and 200 nm. Both the pristine hydrogel and the supernatant were subjected to SEM characterization.

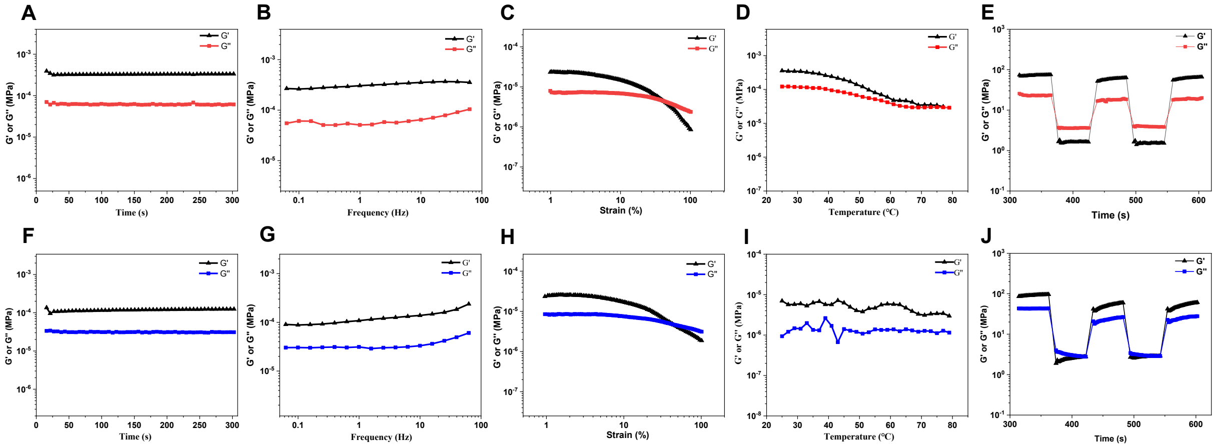

Figure S13. Rheology of Time, dynamic frequency sweep, strain-dependent oscillatory shear, temperature and step-strain. (A-E) Fmoc-*L*-Y/BBR chiral hydrogels; (F-J) Fmoc-*D*-Y/BBR chiral hydrogels.

Time sweep experiments revealed that the storage modulus (G') of the Fmoc-Y/BBR chiral hydrogels were always higher than the loss modulus (G"), confirming excellent elastic ability over an extended period Dynamic frequency sweep results indicated that within the frequency range of 0 to 100 Hz, the G' value of the hydrogel was consistently higher than G", highlighting the superior stability of Fmoc-Y/BBR gels. As the oscillatory strain increased, the intersection of G" and G' revealed the hydrogel's shear strain capacity. Temperature rheology analysis showed that when the temperature exceeded 80 ℃, G" surpassed G', indicating the change from gel state to solution state. It indicated that Fmoc-Y/BBR gels were stable. Subsequently, the self-healing properties of the Fmoc-Y/BBR gels were verified through step-strain tests. The experiments revealed that the Fmoc-Y/BBR gels maintained their solid structure under a low strain of 0.1%. However, when the strain was increased to 300%, their storage modulus (G′) plummeted, resulting in the disintegration of the gel network and a transition to the sol state. The results obtained from repeated cycles were highly consistent with the first cycle, unequivocally demonstrating the rapid and complete self-healing capability of the material. In summary, the above rheological results indicated that Fmoc-Y/BBR gels had good self-healing properties, thixotropy, and thermal reversibility, are potential clinical biomaterials.

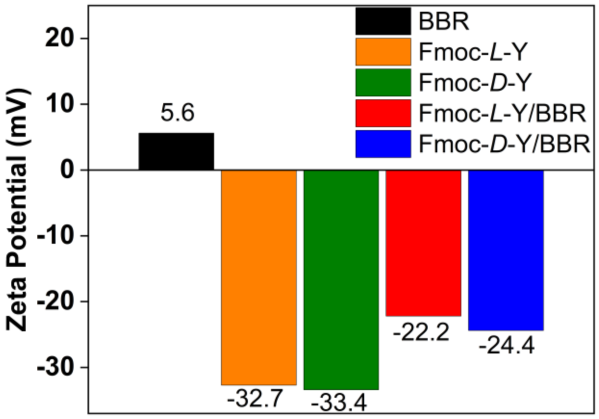

Figure S14. Zeta potential values. All sample tests were conducted at a concentration of 1.73 mM.

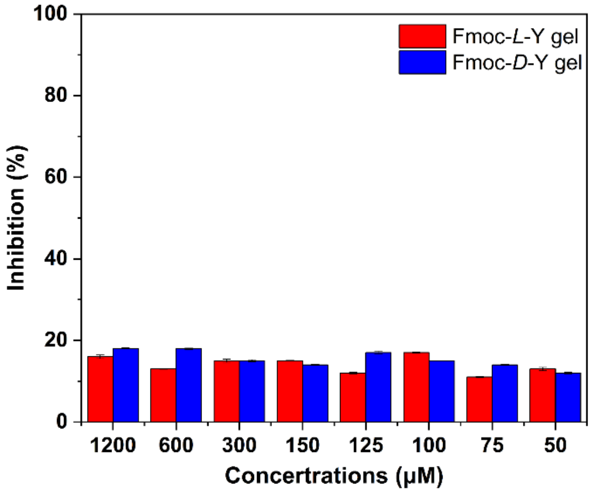

Figure S15. Antibacterial activity of Fmoc-Y self-assemblies.

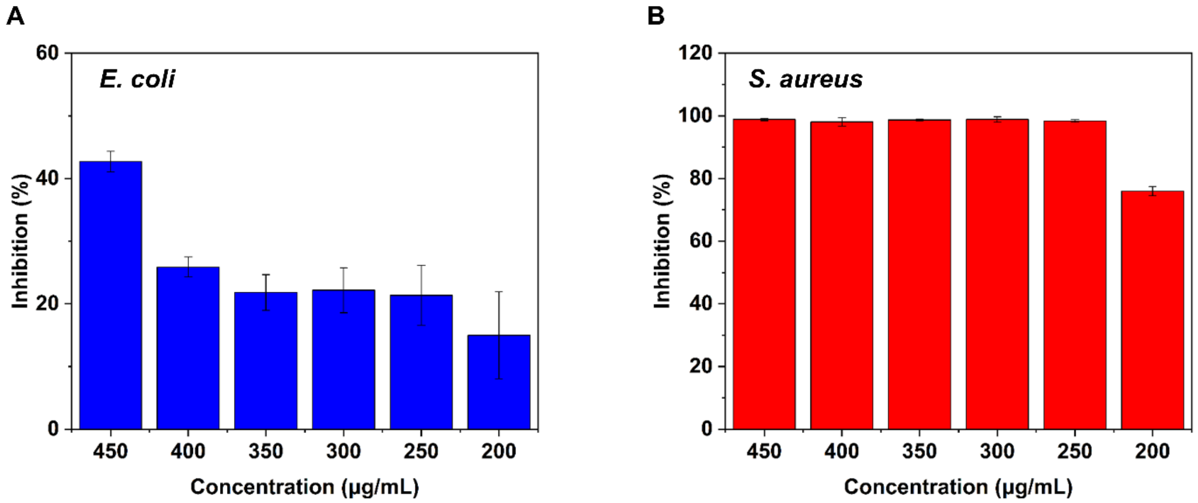

Figure S16. Minimum Inhibitory Concentration (MIC) of Fmoc-*L*-Y/BBR co-assemblies. Bacterial inhibition rate of *E. coli* (A) and *S. aureus* (B). Values are expressed as the means ± SD (n = 3).

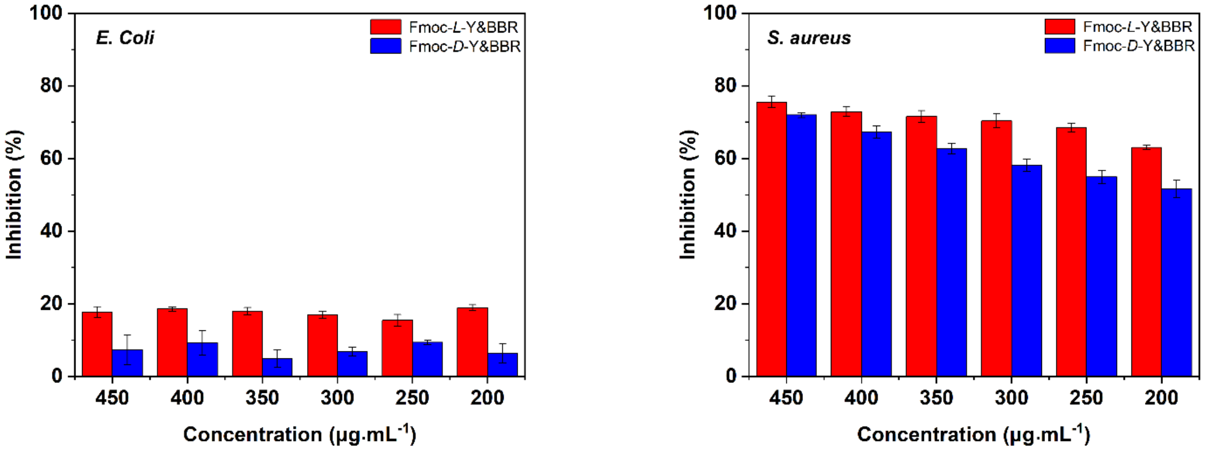

Figure S17. Antibacterial activity of physical mixture of nanofibers with BBR. Values are expressed as the means ± SD (n = 3).

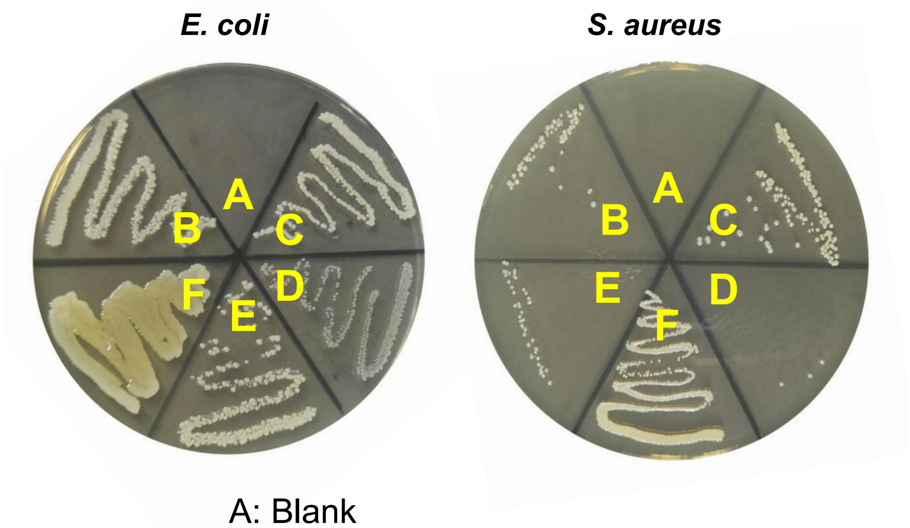

A: Blank
B: Fmoc-*L*-Y/BBR under dark
C: Fmoc-*D*-Y/BBR under dark
D: Fmoc-*L*-Y/BBR with white light
E: Fmoc-*D*-Y/BBR with white light
F: Control

Figure S18. Photographs of agar plate delineations. Incubated under 20 mW·cm^{-2} white light for 1 hour. B-E Samples were treated at 250 μg·mL^{-1} with BBR content at 8.6 μg·mL^{-1}.

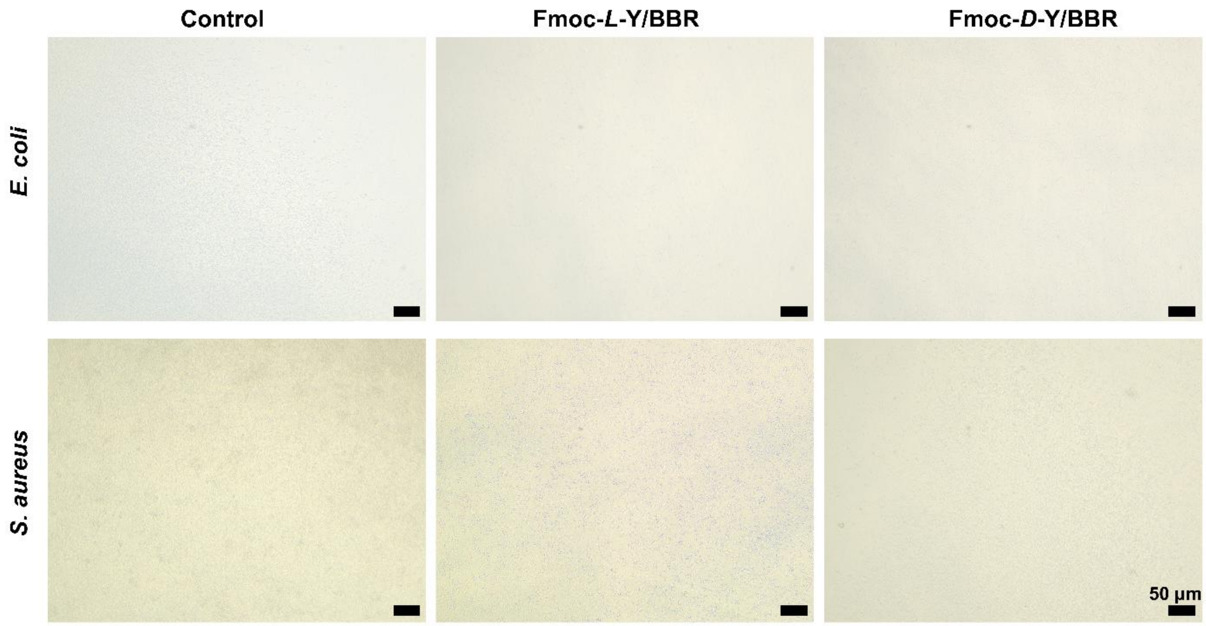

Figure S19. Bright-field images of the bacteria.

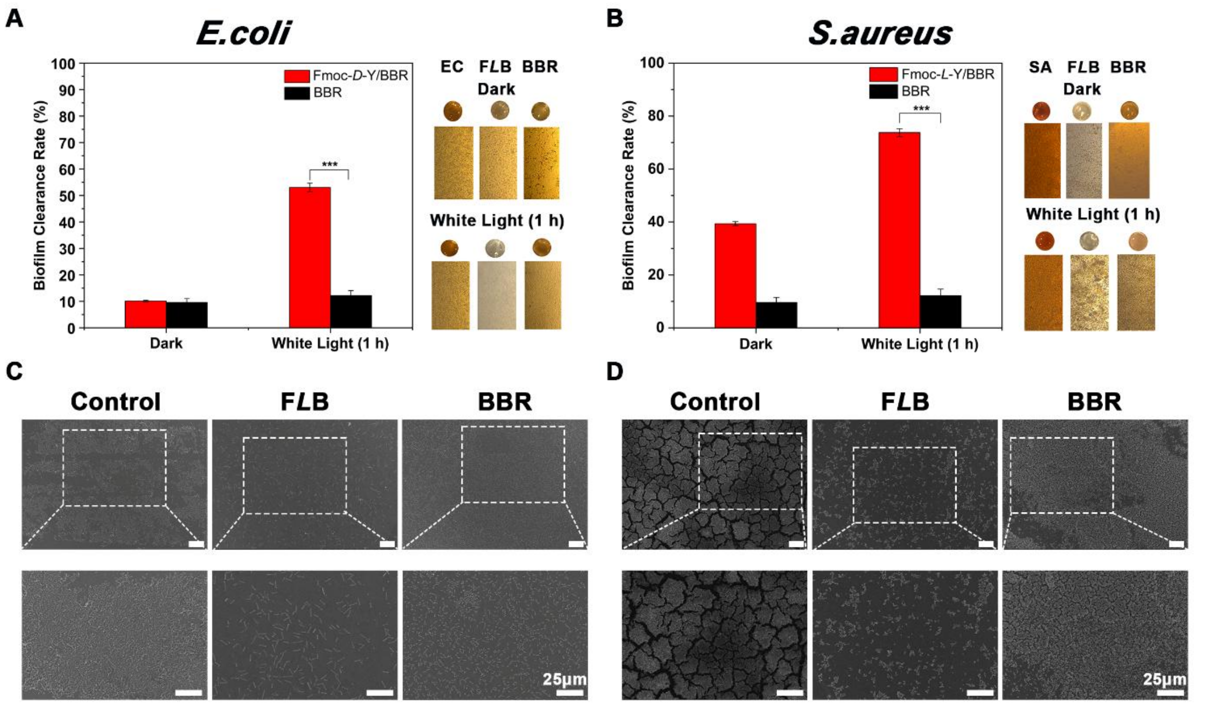

Figure S20. Biofilm clearance assay. (A) XTT assay test of *E. coli*; (B) XTT assay test of *S. aureus*; (C) SEM images of *E. coli* biofilm; (D) SEM images of *S. aureus* biofilm. scale bars: 25 μM. Incubated under 20 mW·cm^{-2} white light for 1 hour. All samples were treated at 250 μg·mL^{-1} with BBR content at 8.6 μg·mL^{-1}. Values are expressed as the means ± SD (n = 3). **p* < 0.05, ***p* < 0.01, and ****p* < 0.001 (*n* = 3).

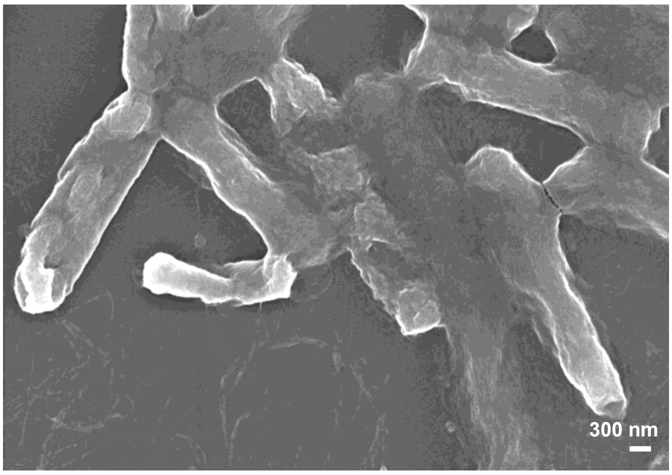

Figure S21. Morphology of *E. coli* cells treated with Fmoc-*L*-Tyr/BBR assemblies. *E. coli* bacterial cells treated with Fmoc-*L*-Y/BBR 250 μg·mL^{-1} with BBR content at 8.6 μg·mL^{-1}. Incubated under 20 mW·cm^{-2} white light for 1 hour; Scale bars: 300 nm.

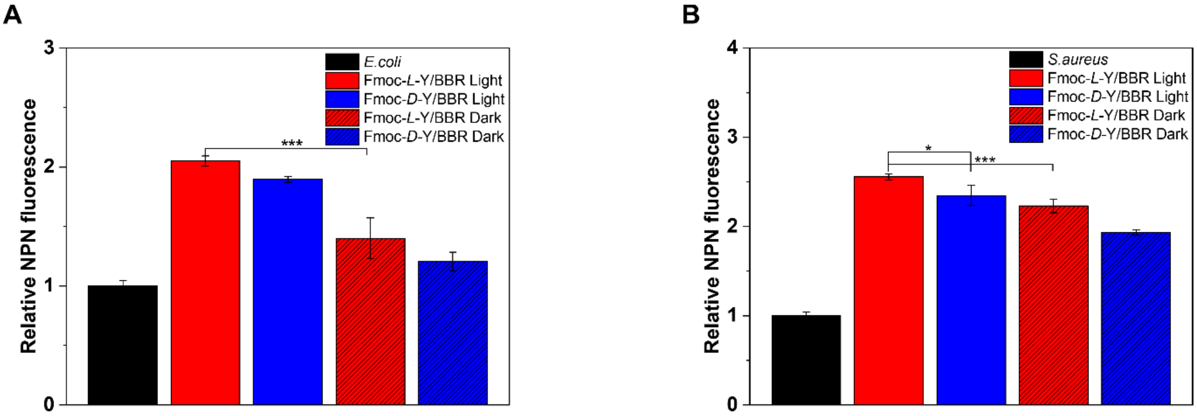

Figure S22. Assessment of outer membrane permeability in *E. coli* (A) and *S. aureus* (B) by the NPN uptake assay. Values are expressed as the means ± SD (n = 3). **p* < 0.05, ***p* < 0.01, and ****p* < 0.001 (*n* = 3).
